# *In Vitro* and *In Vivo* Studies for the Investigation of γ-Globin Gene Induction by *Adhatoda vasica*: A Pre-Clinical Study of HbF Inducers for β-Thalassemia

**DOI:** 10.3389/fphar.2022.797853

**Published:** 2022-03-29

**Authors:** Fizza Iftikhar, Saeedur Rahman, Muhammad Behroz Naeem Khan, Kanwal Khan, Muhammad Noman Khan, Reaz Uddin, Syed Ghulam Musharraf

**Affiliations:** ^1^ Dr. Panjwani Center for Molecular Medicine and Drug Research, International Center for Chemical and Biological Sciences, University of Karachi, Karachi, Pakistan; ^2^ H.E.J. Research Institute of Chemistry, International Center for Chemical and Biological Sciences, University of Karachi, Karachi, Pakistan

**Keywords:** erythroid differentiation, bioassay-guided isolation, Adhatoda vasica, pyrroquinazoline alkaloids, hematological indices, HbF induction, biochemical and hematological studies

## Abstract

Fetal hemoglobin (HbF) is a potent genetic modifier, and the γ-globin gene induction has proven to be a sustainable therapeutic approach for the management of β-thalassemia. In this study, we have evaluated the HbF induction ability of *A. vasica in vitro* and *in vivo*, and the identification of potential therapeutic compounds through a bioassay-guided approach. *In vitro* benzidine-Hb assay demonstrated strong erythroid differentiation of K562 cells by *A. vasica* extracts. Subsequently, an *in vivo* study with an aqueous extract of *A. vasica* (100 mg/kg) showed significant induction of the γ-globin gene and HbF production. While in the acute study, the hematological and biochemical indices were found to be unaltered at the lower dose of *A. vasica*. Following the bioassay-guided approach, two isolated compounds, vasicinol (**1**) and vasicine (**2**) strongly enhanced HbF levels and showed prominent cellular growth kinetics with ample accumulation of total hemoglobin in K562 cultures. High HbF levels were examined by immunofluorescence and flow cytometry analysis, concomitant with the overexpression in the γ-globin gene level. Compound **1** (0.1 µM) and compound **2** (1 µM) resulted in a greater increase in F-cells (90 and 83%) with marked up (8-fold and 5.1-fold) expression of the γ-globin gene, respectively. Molecular docking studies indicated strong binding affinities of (**1**) and (**2**) with HDAC2 and KDM1 protein that predict the possible mechanism of compounds in inhibition of these epigenetic regulators in the γ-globin gene reactivation. Altogether, these observations demonstrated the therapeutic usefulness of *A. vasica* for fostering HbF production in clinical implications for blood disorders.

## Introduction

β-Thalassemia is an inborn monogenic defect of β-globin production in hemoglobin leading to excess accumulation of α-globin chain that affects membrane integrity of RBCs, causes premature death of erythroid cells, and results in chronic anemia. The mainstay of treating thalassemia includes lifelong blood transfusion and iron chelation (Galanello and Origa, 2010). At present, bone marrow transplantation and gene therapy have also offered great curative potential in its treatment, but their clinical utility is limited. Moreover, the unavailability of these modalities to the common person in the least developed countries is also a pressing problem (Strocchio and Locatelli, 2018; Karponi and Zogas, 2019; Badawy et al., 2021).

Fetal hemoglobin (HbF, α2γ2) plays an important role as a physiological substitute of hemoglobin in normal adult erythrocytes of β-thalassemia patients. So, revitalizing a fetal type of hemoglobin offers transfusion-free treatment and a better quality of life to thalassemic patients (Cui and Engel, 2017; Sun et al., 2022). Various epigenetic regulators (hydroxyurea, 5- azacytidine, short-chain fatty acid derivatives, and butyrate) have provided attractive therapeutic targets in γ-globin reactivation with diverse mechanistic actions such as HDAC inhibition, DNA methyltransferases (DNMT), LDS1/KDM1 inhibition, and enhanced transcriptional activity at DNA (Cao et al., 2004; Fathallah et al., 2007; Musallam et al., 2013; Rivers et al., 2016; Gilmartin et al., 2020). At present, the FDA-approved HbF inducer hydroxyurea has shown varying responding rate in a specific population of thalassemia; furthermore, its cytotoxic nature, infertility, and marrow suppressive effect has also led to health concerns (Strouse et al., 2008; Algiraigri et al., 2017).

Natural products are historically proven therapeutic agents in β-hemoglobinopathies. Importantly, the utilization of natural HbF inducers in countries with a high prevalence rate of disease is more affordable as compared to other ways of treatment. Some reported HbF inducers from natural sources are resveratrol, mithramycin, rapamycin, curcuminoid, cucurbitacin D, cinchonidine, and quinidine (Liu et al., 2010; Ng and Ko, 2014; Iftikhar et al., 2019; Iftikhar et al., 2020; Zuccato et al., 2021). Yet, many of these are recently identified HbF inducing agents, and their therapeutic applications are restricted due to their carcinogenic and cytotoxic properties and require further clinical trials (de Dreuzy et al., 2016). Therefore, the need for new HbF inducers especially from medicinal plants exemplified with enhanced efficacy and minimal side effects is highly sought after.

*Adhatoda vasica* Nees also known as *Justicia adhatoda* is a small evergreen medicinally important plant, commonly found in many areas of Pakistan and globally (Rahman et al., 2019). *Adhatoda vasica* (*A. vasica*) is well-known in unani and ayurveda and has been extensively used to treat upper respiratory tract ailments. Other reported pharmacological activities of *A. vasica* are antibacterial, antidiabetic, hepatoprotective, anti-allergy, anti-inflammatory, antitumor, antimalarial, and antifungal activities (Gangwar and Ghosh, 2014; Hossain and Hoq, 2016; Nikhitha et al., 2021; Shamsuddin et al., 2021; Shoaib, 2021). The primary alkaloid of *A. vasica* (vasicine and vasicinone) are well-known therapeutic respiratory agents. Vasicine has also been known for its diverse biological attributes including anti-inflammatory, anti-cancer, anti-hypertensive, abortifacient, anti-allergic, and anti-tubercular activities (Singh and Sharma, 2013; Ghanta et al., 2020; Kumari et al., 2021). The well-established chemical and pharmacological potential of *A. vasica* for the treatment of various ailments prompts us to investigate and identify potent HbF inducers from this ethnomedicinal plant and repurpose its use as HbF inducers for β-thalassemia treatment.

## Methodology

### K562 Culture Conditions and Benzidine–H_2_O_2_ Assay

Human erythroleukemia K562 cells (CCL-243) were purchased from American Type Culture Collection (ATCC) and grown in RPMI-1640 medium (Gibco BRL, United States), supplemented with 10% fetal bovine serum (FBS) (Sigma, United States), and 1% penicillin/streptomycin (Sigma, United States) as described previously (Iftikhar et al., 2019). A stock solution of 10 mg/ml (for extract and fractions) and 20 mM (for pure compounds) was prepared and stored at −20°C, which was diluted afterward during experimentation. Hydroxyurea (Sigma Aldrich, United States) at 200 μM was used as the positive control, and untreated cells were used as the negative controls. Cells were seeded in a 96-well plate at a density of 4 × 10^3^ cells per mL, treated with different concentrations of extracts (0.1–500 μg/ml) and pure compounds in a concentration range of 0.1–200 µM under a humidified atmosphere with 5% CO_2_ at 37°C for 5 days.

For benzidine–H_2_O_2_ staining in K562 cultures, treated cells were washed with PBS solution and suspended in 0.2% benzidine solution (benzidine hydrochloride in 0.5 M glacial acetic acid with 10% H_2_O_2_) for 5 min in dark (Iftikhar et al., 2019). Erythroid differentiation was examined by counting benzidine-positive (HbF-containing) cells and photographed by using an inverted microscope (Micros, Austria).

### *In vivo* Study Using Transgenic Mice and Ethical Approval

The *in vitro* erythroid differentiation ability of *A. vasica* was further validated by using β-YAC transgenic mice to assess induction and γ-globin gene expression. The transgenic mice containing the human β-globin gene locus including the locus control region (LCR) in a yeast artificial chromosome (YAC) was used with the approval of the Institutional Animal Care and Use Committee of the Animal Research Facility (Animal Safety Protocol number 2017-0003) (Boosalis et al., 2015; Iftikhar et al., 2020). These β-YAC mice were already characterized previously (Costa et al., 2012) and bought from the University of Kansas Medical Center. Animals were bred and housed in sawdust containing cages under controlled conditions of 12-h light–dark cycles, humidity (40–70%), and temperature (22 ± 2°C). They were provided with standard mice feed and water ad libitum in a room established at the animal facility of the university. Genotyping of tail biopsies was performed by using conventional PCR. Primer sequences used to identify the murine alpha hemoglobin as the internal control and the human γ-globin genes are listed in Table 1.

**TABLE 1:** Sequences of primers used in qRT-PCR analysis and genotyping of transgenic mice.

Gene	Primer	Sequences (5′→3′)	Size (bp)
Primers for qRT-PCR analysis			
Human γ-globin	Forward	TTCCTGGCAGAAGATGGT	82
	Reverse	AGC​TCT​GAA​TCA​TGG​GCA​GT	
Human GAPDH	Forward	CCA​GAA​CAT​CAT​CCC​TGC​CT	120
	Reverse	CCT​GCT​TCA​CCA​CCT​TCT​TG	
Mice GAPDH	Forward	GTA​TGA​CTC​CAC​TCA​CGG​CA	147
	Reverse	TCC​ACG​ACA​TAC​TCA​GCA​CC	
Primers for genotyping of transgenic mice			
Mouse α-hemoglobin	Forward	ACT​AAC​TTC​TTC​CCA​AAC​TGC​CAT​CA	213
	Reverse	AAG​GGC​TGT​CCT​CCA​GGC​AGG​GTG​G ACAC	
Human γ-globin	Forward	TAT​CTG​TCT​GAA​ACG​GTC​CC	275
	Reverse	CCA​CAG​GCT​TGT​GAT​AGT​AG	

### Treatment Protocol and Analysis for *In Vivo* Study

For treatment, β-YAC transgenic mice 6–8 weeks old were grouped (N = 5). Animals were tested for HbF induction ability by acute and chronic IP administration of aqueous extract of *A. vasica* at different doses, with hydroxyurea at (200 mg/kg/bodyweight) as positive controls. *A. vasica* (aqueous extract) was prepared in sterile, neutral pH DI water. Water was used as the vehicle (control). For acute HbF induction, mice were grouped (N = 5) and an aqueous extract of *A. vasica* was administered *via* two different doses of intraperitoneal injection at 5 mg/kg/bodyweight and 60 mg/kg/bodyweight, respectively, for 15 consecutive days.

For a prolonged HbF potential effect of *A. vasica*, mice were dosed intraperitoneally at various doses (100, 200, and 500 mg/kg/bodyweight) for 4 weeks. Following post-treatment, mice were sacrificed, and blood was collected for monitoring the F-cell population by flow cytometry. Bone marrow was flushed from the femurs and processed for total RNA extraction to evaluate γ-globin gene expression by using the qRT-PCR platform as described in the experimental section of mRNA analysis of γ-globin gene and analysis of HbF expression.

Acute toxicity studies were conducted in wild-type male and female groups of mice under the guidelines of the Organisation for Economic, Co-operation, and Development (OECD) 45 Test to determine the median lethal dose (LD50). In another set of experiments, C57BL/6 mice were orally administrated with an aqueous extract of *A. vasica* at various doses (100, 200, and 500 mg/kg). Post-treatment, mice were sacrificed, and blood samples were collected analyzed for hematological and serum biochemical indices as described elsewhere (Silva-Santana et al., 2020).

### Extraction and Purification of Compounds From *A. vasica*

The whole plant of *A. vasica* was collected from district Bajaur, Khyber Pakhtunkhwa, Pakistan, during May 2019, identified, and allotted a voucher specimen (No. KUH 53882) by the University of Karachi. The dried plant material (approx. 2.50 kg) was extracted using 50% methanol in water at room temperature to obtain a dried crude extract (approx. 300 g). Following bio-assay guided isolation, the crude extract was then subjected to solvent–solvent partitioning using n-hexane, chloroform, and ethyl acetate. Afterward, the extract was first eluted with 100% deionized water and then different isocratic systems with 10, 30, 50, 70, and 100% MeOH:H_2_O were used to obtain six fractions (F1–F6). Based on HbF induction activity, 30% methanol fraction (F3) was further subjected to the isolation of compounds.

Isolation and purification of compounds **1**–**4** were carried out by using VLC and recycling preparative HPLC ((LC-908), respectively. VLC was used for fractionation by using silica gel (C18-reverse phase, Merck). The infrared (IR) spectra of purified compounds **1**–**4** were recorded on an FT-IR machine (Shimadzu-8900, Japan). ^1^H and ^13^C-NMR spectra were recorded on Bruker Avance-NMR (400,500 and 600 MHz) instruments using CD_3_OD as a solvent. Multiplicities of carbon signals were defined by using DEPT-90° and 135° experiments. Mass spectrometer (JMS 600H) coupled with TSS 2000 was used to record the EI-MS spectra. HR-ESI-MS spectra of compounds **1**–**4** were recorded on a mass spectrometer (ESI-QTOF, Bruker Maxis II™, Germany). HPLC-DAD profiles of the crude extract, mostly of the active fraction (F3), and purified compounds (**1**-**4**) were achieved by using an HPLC system (Agilent technologies-1260 infinity, Germany) fitted with a C18 (3 × 100 mm, 1.8 µm) column (Macherey-Nagel, Nucleodur gravity, Germany) using a gradient elution of H_2_O/MeOH. All solvents and chemicals used in this work were of analytical grade.

### Effects of Compounds on Erythroid Differentiation, Cellular Growth, and Cytotoxic Study

The erythroid differentiation ability of purified compounds (**1**, **2**, **3**, and **4**) from *A. vasica* were examined by benzidine-Hb assay as described previously. Briefly, induction was carried out in a concentration-dependent style for all the four compounds, and benzidine-positive cells indicated the induction of intracellular Hb, observed by light microscopy.

The antiproliferative effect of lead compounds was examined by treating K562 cells with different concentrations (0.1–25 µM) of lead compounds for 144 h. Cellular growth was evaluated by determining the viable cells/mL after the incubation period using trypan blue dye exclusion assay. The cytotoxic effects of lead compounds were determined using alamarBlue (AB) calorimetric assay as reported previously (Iftikhar et al., 2019). The mean fluorescence was measured at the respective excitation and emission wavelength of 560 and 590 nm using a Spectramax M5e multi-mode microplate reader (Molecular Devices, United States) with Softmax pro software (Molecular Devices, United States). Results were expressed as the percent control of viable cells, and IC_50_ was calculated.

### Total Hemoglobin Synthesis Determination

The quantitative amount of total hemoglobin synthesized after treating the cells with lead compounds was assessed using the plasma hemoglobin kit (Sigma, United States), as reported previously (Iftikhar et al., 2020). K562 cell lysate was obtained by using lysis buffer containing 0.2% Triton X-100 in 100 mM potassium phosphate (pH 7.8). The lysate was then centrifuged, and 50 µL of supernatant was incubated with a reagent mixture provided with a kit for 5 min. The optical density of the samples was measured at 400 nm using a microplate reader (Spectramax, United States). The hemoglobin content (pg/cell) was calculated and represented as the mean ± SEM of three independent experiments.

### mRNA Analysis of the γ-Globin Gene by Real-Time Quantitative PCR (qPCR*)*

To analyze the γ-globin gene expression, real-time PCR was performed on RNA samples from treated K562 cells and post-treated murine-derived bone marrow. Briefly, K562 cells (approximately 1 × 10^5^ cells) were induced with different concentrations of lead compounds in comparison to HU-200 µM and untreated controls. The TRIzol extraction method was used for RNA extraction and purified using a DNase 1 kit (EN0521, Thermo Scientific Baltics, UAB). RNA samples were subjected to cDNA synthesis for 1 h at 42°C *via* a RevertAid first-strand cDNA synthesis kit (Thermo Scientific Baltics, UAB). The obtained cDNA was used as a template, and qRT-PCR was performed using a Maxima SYBR Green/ROX qPCR Master Mix (2X) (K0221, Thermo Scientific Baltics, UAB) on an Mx3000P QPCR System (Agilent Technologies, United States) as previously described conditions (Aimola et al., 2016). The expression of the gene was evaluated using the 2^−ΔΔCt^ method and normalized by using the reference GAPDH gene. All the primers for real-time PCR are documented in Table 1.

### Analysis of HbF Expression by Immunofluorescence Microscopy and Flow Cytometry

K562 cultures were examined for HbF expression analysis by using immunofluorescence staining and flow cytometry. For immunofluorescence analysis, treated cells were first fixed with 4% PFA for 20 min and permeabilized with 0.1% Triton X-100 for 5 min, while momentarily being washed with PBS thrice after each step. For cell staining, overnight incubation with PE-conjugated anti-HbF antibodies (MHFH04; Life Technologies, Frederick) at dilution of 1:200 was performed. The microscopic images were acquired by using an immunofluorescent microscope (Nikon Eclipse 90i, Japan) using NIS-Elements software (Nikon, Japan). The quantified HbF-positive cells are represented as the mean fluorescence intensity/cell computed by using ImageJ software ver. 1.5 (NIH, United States).

For Flow analysis, a previously described method was followed on differentiated K562 cells and mice whole blood (Iftikhar et al., 2020). Briefly, treated cells were fixed and permeabilized with 0.05% glutaraldehyde and 0.1% Triton X-100, respectively. The cells were labeled with a PE-conjugated mouse anti-human fetal hemoglobin antibody for 20 min in dark at room temperature. After washing with 0.1% BSA/PBS, cells were analyzed on a flow cytometer (BD FACSCanto II, United States) through a PE channel using BD CellQuest Pro software (BD Biosciences, United States). HbF-positive cells were measured and expressed as a percentage of F-cells.

### Molecular Docking Studies to Target the Epigenetic Regulators Involved in γ-globin Gene Expression

To evaluate the possible epigenetic mechanism involved in the HbF induction by lead candidates at the γ-globin gene (*HBG1* and *HBG2*) locus, the interaction of compounds **1** and **2** (ligands) with repressing transcriptional switch, BCL11A (drug target), and epigenetic enzyme complexes (DNMT1, KDM1, HDAC1, and HDAC2) recruited by BCL11A were studied (Saunthararajah, 2019). The identification of the active site of these enzymes is provided in the Supplementary Material (Choubey and Jeyaraman, 2016; Abdulazeez, 2019; Alsawalha et al., 2019). Docking studies were performed using the PDB database, AutoDock 4.2, Pose View, and ezCADD. The STRING database is used to highlight the interaction of γ-globin gene suppressor (epigenetic enzymes) and to validate their function and interaction (Szklarczyk et al., 2016). The data retrieval and its validation process are provided in the Supplementary Material.

### Statistics

Statistical analysis was performed using GraphPad Prism 6.0 software (GraphPad, United States). Quantitative data obtained from compounds and treated cultures were compared to the untreated control. Comparison analysis was performed using Dunnett’s multiple comparison test or student’s t-test. *p < 0.05 was considered statistically significant.

## Results

### Erythroid Differentiation Effects of *A. vasica* on K562 Cells

**FIGURE 1:**
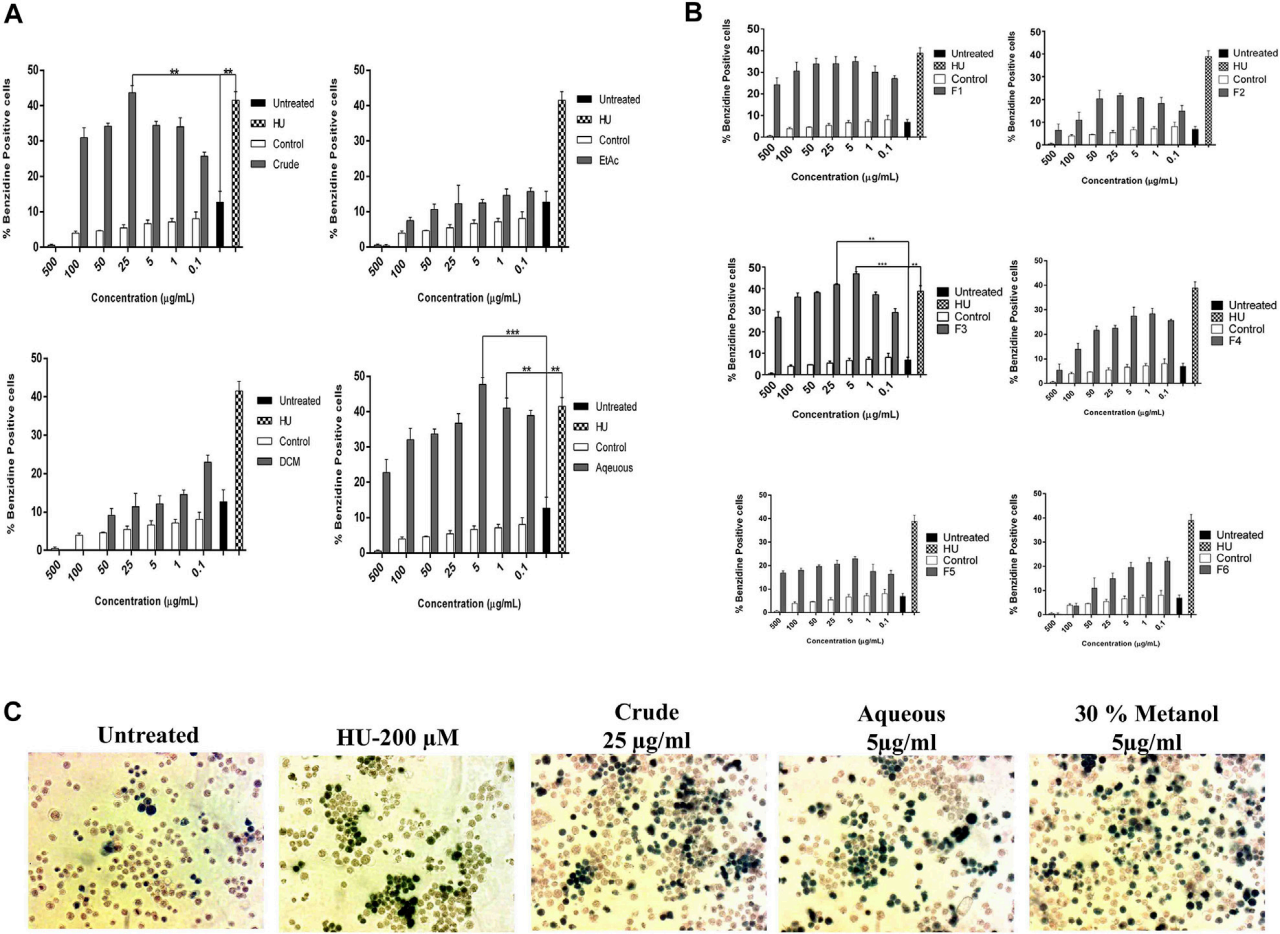
Bioassay-guided activity of *A. vasica* on erythroid differentiation of K562 cells assayed by benzidine–Hb staining. **(A)** The HbF induction activity of the crude, DCM, EtOAc, and aqueous extracts. **(B)** K562 cells were treated with six sub-fractions (F1–F6) for 5 days to evaluate the inducing effects. Data is obtained from three independent experiments in triplicate and shown as mean ± SEM; *p < 0.05 *vs*. control. **(C)** Microscopic images of treated cultures showed differentiating benzidine-positive (induced cells) uninduced K562 cells.

Initially, the erythroid differentiation ability of *A. vasica* was examined in K562 cells, followed by bioassay-guided isolation. Preliminary studies with a crude extract in a dose-dependent manner demonstrated the potent erythroid differentiation ability of *A. vasica.* The differentiation rate (43.67 ± 2.01%) by crude extracts at 25 μg/ml showed maximum erythroid differentiation (Figure 1A). Subsequently, treatment with aqueous, dichloromethane (DCM) and ethyl acetate (EtOAc) extracts at different inducing dosages demonstrated that aqueous extracts were more potent for their ability to induce fetal hemoglobin (Table 2). The graphical representation describes that aqueous extracts in a concentration range of 0.1–25 μg/ml showed the highest induction ability as compared to DCM and EtOAc extracts. The percentages of benzidine–positive cells induced by these extracts at different inducing concentrations are represented in Figure 1A. Successively, erythroid induction was examined by treating K562 cells with different methanol fractions as represented in Figure 1B. Benzidine-staining results demonstrated that among six fractions with different methanol concentrations (F1–F6; 10–100%), a fraction containing 30% methanol (F3) showed significant erythroid differentiation as compared to the untreated culture. The erythroid differentiation rate was measured by counting percentages of benzidine-positive, induced cells in comparison to non-induced cells as shown in microphotographs (Figure 1C).

**TABLE 2:** Erythroid differentiation effects of the crude extract and methanol fraction of A. vasica in a dose-dependent manner, with untreated culture as a negative control and HU as a positive control.

Extracts	Concentration (µM)						
	500	100	50	25	5	1	0.1
Untreated	12.68 ± 3.11	—	—	—	—	—	—
Hu-200	41.51 ± 2.48	—	—	—	—	—	—
Crude (50%) methanol	0.00	30.97 ± 2.73	34.18 ± 0.85	43.67 ± 2.01	34.45 ± 1.12	34.06 ± 2.53	25.75 ± 1.10
EtOAc	0.37 ± 0.17	7.46 ± 0.94	10.66 ± 1.50	12.26 ± 5.20	12.48 ± 0.93	14.59 ± 1.86	10.99 ± 5.7
DCM	0.00	0.00	9.14 ± 1.76	11.36 ± 3.43	12.09 ± 2.13	14.55 ± 1.17	22.95 ± 1.83
Aqueous	22.77 ± 3.69	32.06 ± 3.23	33.68 ± 1.40	36.71 ± 2.71	47.70 ± 1.96	40.97 ± 2.86	38.91 ± 1.44
F1	24.31 ± 3.12	30.59 ± 3.95	33.88 ± 2.56	38.00 ± 3.29	36.99 ± 2.12	30.04 ± 2.85	27.09 ± 1.33
F2	6.43 ± 2.75	10.90 ± 3.55	20.31 ± 3.74	21.68 ± 0.91	20.68 ± 0.19	18.23 ± 2.68	14.88 ± 2.46
F3	26.59 ± 2.63	36.08 ± 1.87	38.14 ± 0.44	41.91 ± 0.48	46.93 ± 0.86	37.19 ± 1.19	28.94 ± 1.74
F4	5.36 ± 2.49	13.90 ± 2.42	21.64 ± 1.73	22.51 ± 1.06	27.46 ± 3.57	28.40 ± 2.14	25.59 ± 0.48
F5	16.82 ± 0.94	18.01 ± 0.88	19.66 ± 0.62	20.68 ± 1.57	22.91 ± 0.95	17.52 ± 3.04	16.34 ± 1.53
F6	0.27 ± 0.47	3.60 ± 1.14	10.96 ± 4.23	14.93 ± 2.21	19.55 ± 2.03	21.59 ± 1.93	22.08 ± 1.47

### *In Vivo* Studies Reveal That *A. vasica* Induces HbF Production and γ-globin Transcription in β-YAC Transgenic Mice

**FIGURE 2:**
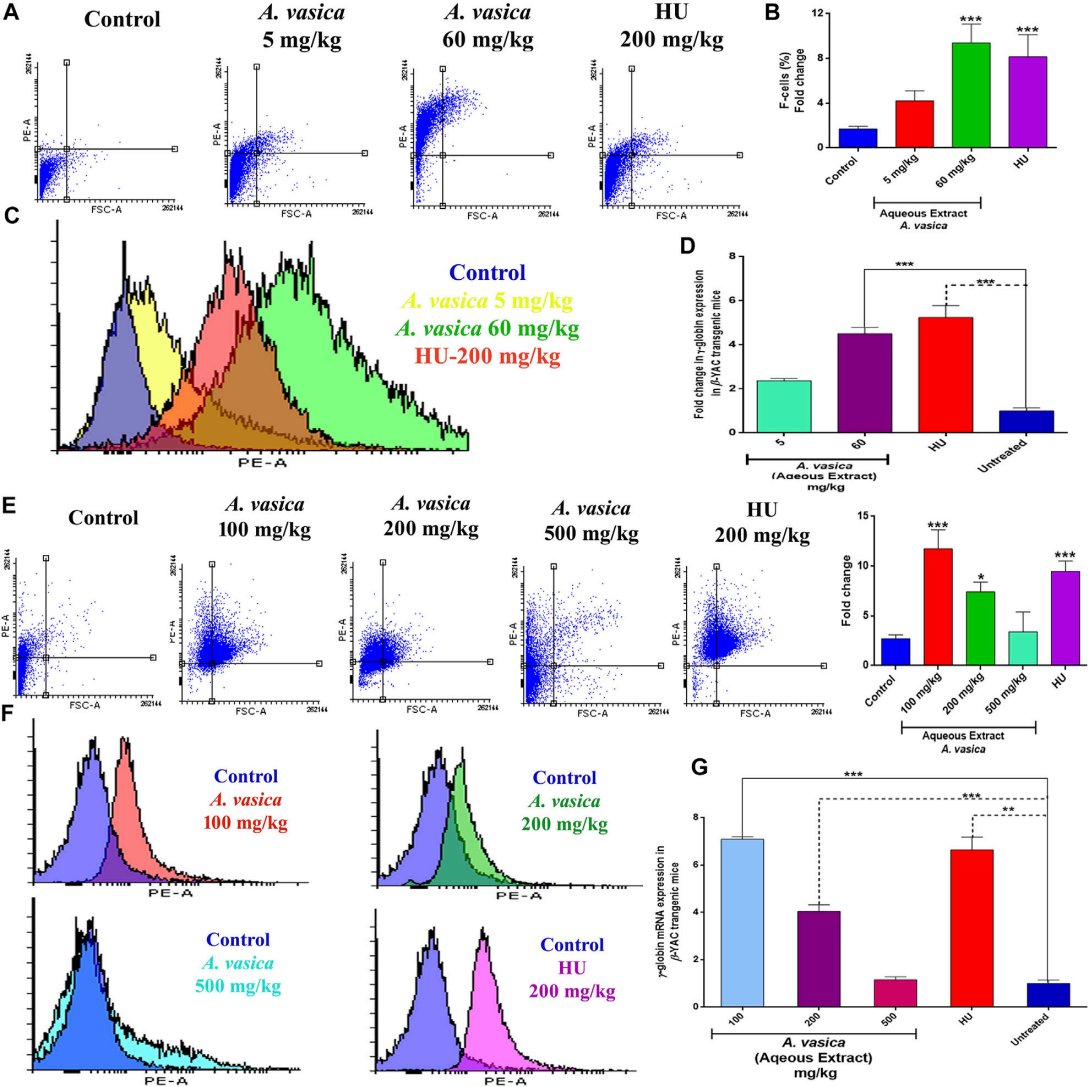
Aqueous extract of *A. vasica* induces HbF production and γ-globin gene expression in β-YAC transgenic mice. **(A)** Preliminary administration of the aqueous extract of *A. vasica* (5 and 60 mg/kg) to mice for 15 days showed HbF-positive cells stained with anti-HbF antibodies observed through the PE channel as represented in dot plots. **(B)** Quantitative representations of induced HbF levels are shown in the bar graph as percent fold change in F-cells. **(C)** The histogram demonstrated the level of F-cells (HbF-containing cells) in comparison to the control. **(D)** The bar graph demonstrated the comparative analysis of γ-globin gene expression in mice treated with the aqueous extract of *A. vasica* vs. the untreated control. **(E)** The intraperitoneal injection of 100, 200, and 500 mg/kg of *A. vasica* were administered to mice for 4 weeks. HbF-positive (F-cells) were analyzed by flow cytometry as represented in dot plots and quantitative representation in bar graph representing the percent fold change in F-cells. **(F)** The overlay histograms of cell population stained positive with anti-HbF antibodies in comparison to control. **(G)** The relative fold change of γ-globin gene expression in mice after treatment is shown in the bar graph at distinct doses of the aqueous extract of *A. vasica*. HU (200 mg/kg) and untreated control were taken as controls. The fold change is represented as 2^−ΔΔCt^. All data are shown as the mean ± SEM (N = 5 per group), and *p < 0.05 was considered statistically significant.

To reciprocate *in vitro* effects of *A. vasica* on erythroid differentiation, a preclinical study has been conducted by using β-YAC transgenic mice. The effect of aqueous extracts of *A. vasica* on HbF protein production was analyzed by flow cytometry given at distinct doses to the mice for a 2 week treatment is shown in Figure 2A. FAC results demonstrated that treatment with 60 mg/kg of the administered aqueous extract of *A. vasica* increases the number of F-cells to a maximum of ninefold increase in F-cell population when compared to water-treated controls as shown in Figure 2B. The comparative increase in HbF-positive cells (F-cells) in response to treatment is represented in the overlay histogram (Figure 2C).

To understand the effects of *A. vasica* on γ-globin gene induction, γ-globin mRNA content was quantified using qRT-PCR in mice treated with different doses of *A. vasica* (aqueous extract). Acute treatment of mice with 5 mg/kg and 60 mg/kg of *A. vasica* resulted in enhanced γ-globin gene levels as shown in Figure 2D. An aqueous extract (5 mg/kg) resulted in upregulation of γ-globin gene with 2.37 ± 0.10% fold, and an aqueous extract (60 mg/kg) showed 4.51 ± 0.28 increase while HU showed 5.24 ± 0.05-fold increase in γ-globin gene expression (*p* < 0.001).

For prolonged treatment of 4 weeks, an analysis of HbF expression in the transgenic murine model treated with the aqueous extract of *A. vasica* (100, 200, and 500 mg/kg/bodyweight) and HU (200 mg/kg/bodyweight) in comparison to the control is shown in Figure 2E. *A. vasica* (100 mg/kg) exhibited enhanced levels of F-cells which was 74.74% (11.74-fold) greater than the individual effect of 200 mg/kg of HU exhibiting 71.5% (9.48-fold) production of F-cells. Moreover, *A. vasica* (200 and 500 mg/kg) also showed production of HbF represented by 63.6% (7.40-fold) and 35.2% of F-cells (3.40-fold), respectively, as compared to F-cells of the control (8.26%) (Figure 2F).

Figure 2G also demonstrated that treatment with an aqueous extract of *A. vasica* (100, 200, and 500 mg/kg) for 4 weeks resulted in the overexpression of the γ-globin gene. The mean values of the five animal groups were compared. The results demonstrated that the *A. vasica* aqueous extract (100 mg/kg) resulted in the upregulation of γ-globin gene with 7.09 ± 0.10% fold when compared to the control group. Subsequently, 200 and 500 mg/kg *A vasica* (aqueous extract) enhanced the γ-globin gene with 4.04 ± 0.28% fold and 1.15 ± 0.12 fold, respectively; HU showed a 6.64 ± 0.52-fold increase in γ-globin gene expression (*p* < 0.001). These results have indicated the enhanced level of γ-globin gene expression and HbF production in both K562 cells and murine-derived mRNA following the aqueous extract of *A. vasica* treatment.

### Effects of *A. vasica* on Hematological Parameters and Biochemical Indices

The dose-dependent effect on hematological indices after administration of aqueous extract of *A. vasica* has been evaluated by using a Sysmex XN-550 Hematology Analyzer and reported in Table 3. Mice have been injected with three different doses of the *A. vasica* extract (100, 200, and 500 mg/kg/bodyweight) for 4 weeks. The foremost changes have been observed in the platelets only when compared to the control. However, at higher doses, significant changes in the leucocyte and platelet counts were more prominent, suggesting cumulative toxicity of the extract at doses greater than 200 mg/kg. Apart from the deviation of platelets and WBCs, other hematological parameters were unaffected in all tested doses.

**TABLE 3:** The hematological parameters of mice after 4 weeks of treatment with distinct doses of the aqueous extract of *A. vasica.*

Parameters	Control	100 mg/kg	200 mg/kg	500 mg/kg
RBC (x10^6^/mL)	9.24 ± 0.9	9.25 ± 1.7	9.53 ± 0.4	9.66 ± 1.4
Hb (g/dl)	12 ± 1.4	12.1 ± 0.78	12.1 ± 2.4	12.6 ± 0.8
Haematocrit (%)	45.65 ± 0.8	44.3 ± 0.2	45.2 ± 1.1	47.8 ± 2.5
WBC (x10^3^/uL)	8.38 ± 2.1	7.68 ± 1.6	**6.18 ± 0.2**	**5.68 ± 0.6**
Platelet (x10^3^/uL)	988.5 ± 3.4	1101 ± 2.0	1143 ± 3.4	**1241 ± 0.1**

Statistically significant data and parameters are in bold.

Following treatment with the *A. vasica* extract at various doses (100, 200, and 500 mg/kg/bodyweight) for 4 weeks, biochemical indices have been evaluated by using a Cobas c-311 Biochemistry Analyzer (Roche, United States) and reported in Table 4. The result demonstrated significant changes in biochemical parameters only at higher doses. The liver enzymes AST, ALT, and AP were measured. A noteworthy increase in AP levels was found with 200 and 500 mg/kg of *A. vasica* extracts when compared to the control, and the levels of AST were found to be decreased in 200 and 500 mg/kg treated mice. Similarly, regarding renal functions, the blood urea nitrogen (BUN) test was performed and the serum creatinine level was measured. The results revealed slightly high levels of urea in 200- and 500 mg/kg-treated mice than that of control-treated mice. However, the creatinine levels and physiological parameters (body weight and food consumption) remained unaltered.

**TABLE 4:** The biochemical parameters of mice after 4 weeks of treatment with distinct doses of aqueous extracts of A. vasica.

Parameters	Control	100 mg/kg	200 mg/kg	500 mg/kg
AP (U/L)	62.5 ± 10.6	65 ± 7.6	67.5 ± 0.4	**75 ± 7.4**
AST (U/L)	225 ± 7.0	227.5 ± 3.3	207 ± 2.4	**165 ± 5.1**
ALT (U/L)	32.5 ± 5.8	32.5 ± 3.2	**16.5 ± 4.9**	**3.5 ± 0.7**
BUN (mg/dl)	15 ± 0.1	17.5 ± 3.5	20 ± 0.4	21.2 ± 0.6
Creatinine (mg/dl)	0.195 ± 0.4	0.195 ± 0.2	0.18 ± 0.02	0.18 ± 0.7

Statistically significant data and parameters are in bold.

### Bioassay-Guided Isolation and Characterization of Marker Compounds

**FIGURE 3:**
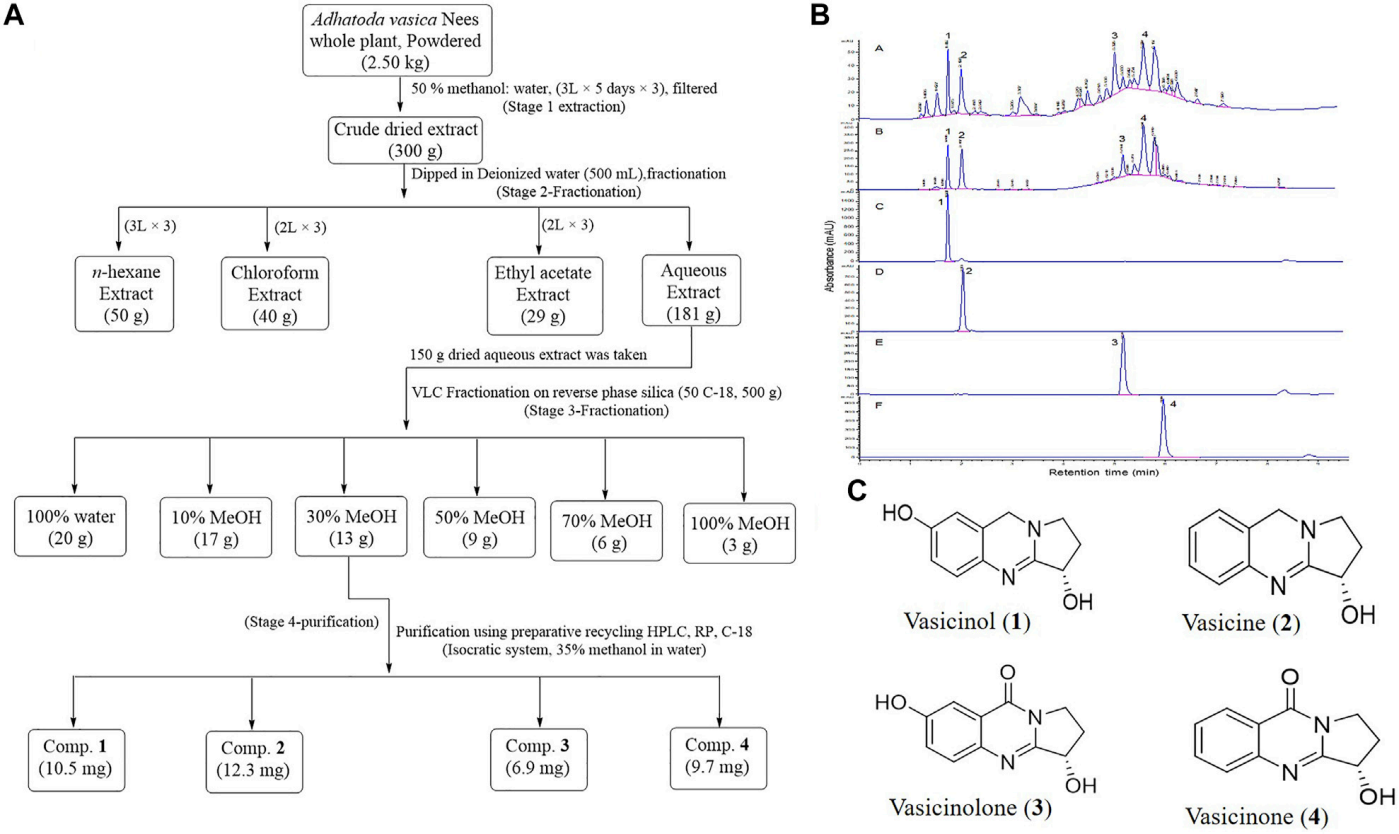
**(A)** Scheme for extraction and purification of compounds **1**–**4**. **(B)** HPLC-DAD profiles and annotation of marker compounds **1**–**4** in crude extracts and the most active fraction F3 (A, B), respectively. HPLC-DAD profiles of purified compounds **1**, **2**, **3**, and **4** are C, D, E, and F, respectively. **(C)** Chemical structures of compounds **1**–**4** purified from the *A. vasica* fraction (F3).

The crude (50% methanol) extract of the whole plant of *A. vasica* showed significant HbF induction activity. Among the solvent–solvent partitioned layers, the aqueous layer showed the strongest HbF induction activity compared to others (Table 2). Among VLC fractions, the 30% methanolic fraction (F3) exhibited the strongest HbF induction potential. The most active fraction (F3) was further purified to obtain pure compounds. The schematic diagram of isolation, purification, and structures of compounds **1**–**4** are shown in Figure 3.

The HR-ESI-MS data of compounds **1**–**4** showed [M + H]+ ions at *m/z* 205.0955, *m/z* 189.1021, *m/z* 2019.0771, and *m/z* 203.0825, which suggested the molecular formulae C_11_H_12_N_2_O_2_, C_11_H_12_N_2_O, C_11_H_10_N_2_O_3_, and C_11_H_10_N_2_O_2_ for compounds **1**, **2**, **3**, and **4**, respectively. The structures of purified compounds were elucidated as vasicinol (**1**), vasicine (**2**), vasicinolone (**3**), and vasicinone (**4**) using different spectroscopic techniques such as NMR, IR, and mass spectrometry. The observed H^1^ and C^13^ NMR spectral data of compounds **1**–**4** are mentioned in Supplementary Table S1. The HPLC-DAD chromatograms of a crude extract, a bioactive fraction (F3), and purified peaks (**1**-**4**) are mentioned in Figure 3B.

**FIGURE 4:**
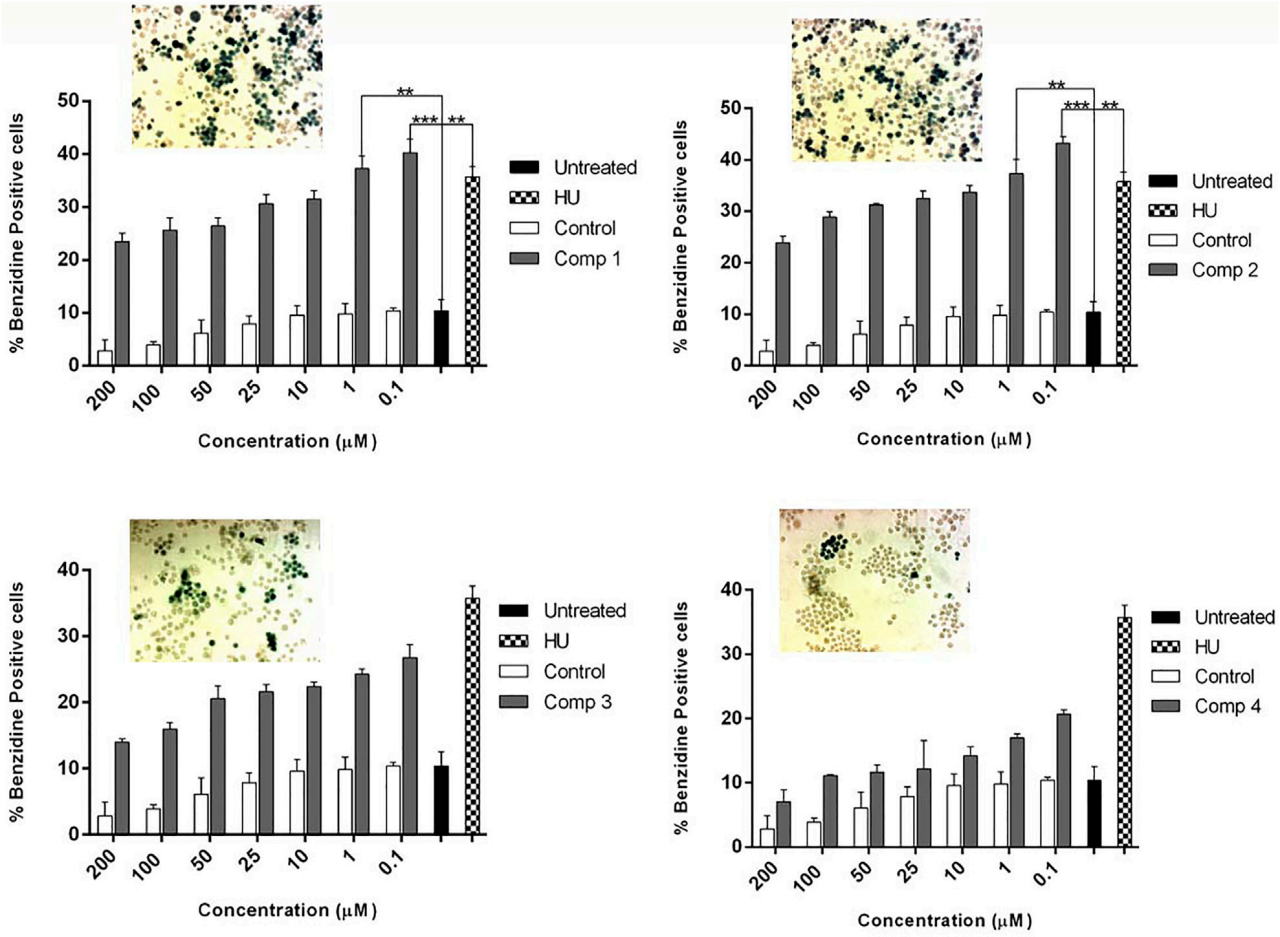
K562 cells were treated with pure compounds of *A. vasica* in a concentration-dependent manner to assess the erythroid differentiation and induction by benzidine–Hb assay. Cells were harvested on day 5 after and the proportion of benzidine-positive (hemoglobin-containing) cells was calculated. Data shown are representative of three independent experiments and represented as mean ± SEM; * *p* < 0.05 was considered statistically significant *vs.* untreated cultures.

Figure 4 demonstrate the dose-dependent erythroid differentiation ability of purified compounds after 5 days of treatment. Comparison of results using different concentrations showed that two of four compounds **1** and **2** were found to be more effective in inducing HbF than HU-200 µM. Compound **1** was found to be most active at 0.1 µM with the rate of benzidine-positive cells as 40.27 ± 2.53, while the optimal concentration of compound **2** was found 1 µM with the rate of benzidine-positive cells as 40.27 ± 2.53.

### Assessment of Cellular Growth and the Cytotoxic Effect, and the Accumulated THb Content

**FIGURE 5:**
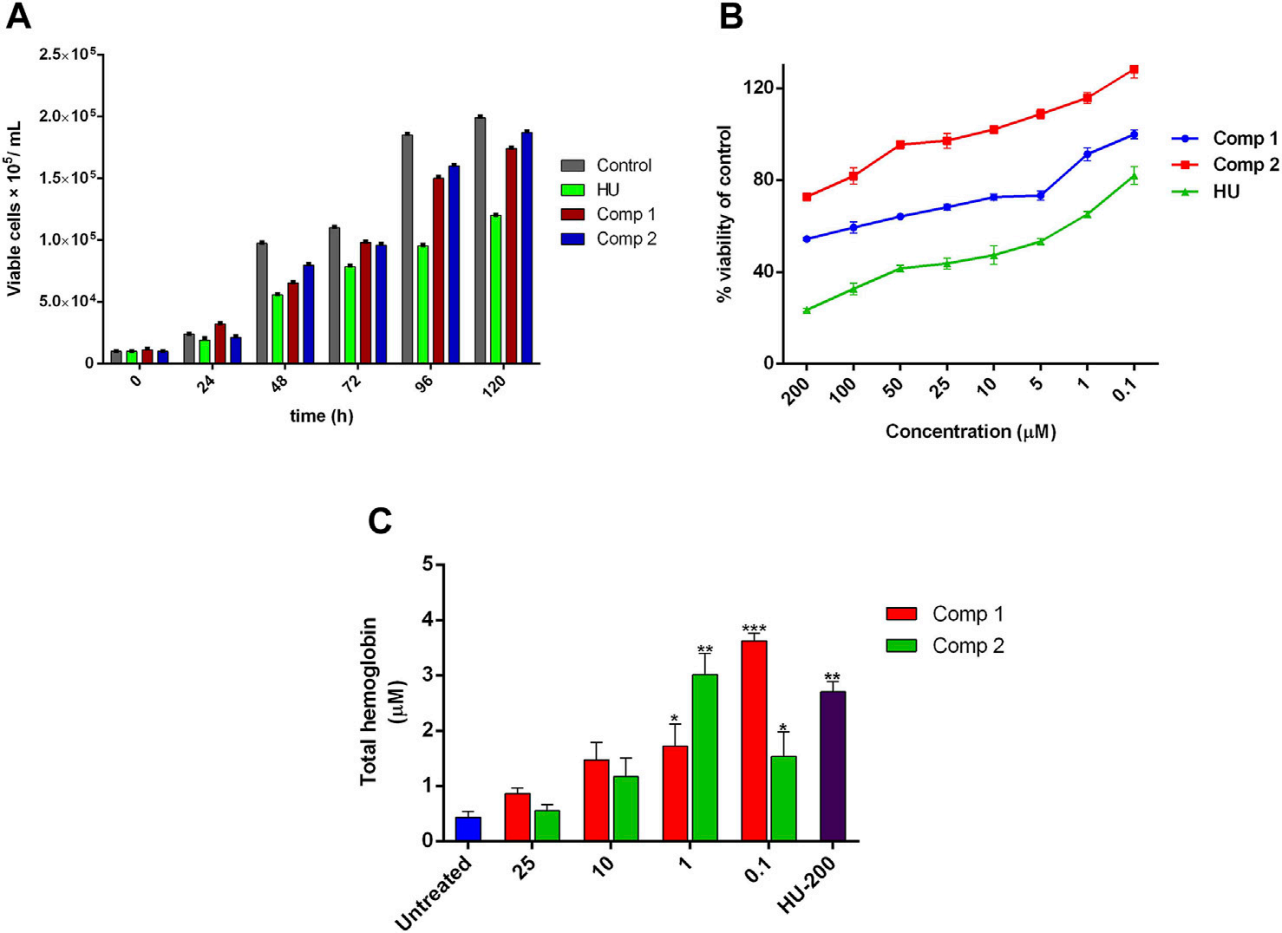
Effect of leads compounds on growth kinetics, cell viability, and THb production in K562 cells. **(A)** The total number of cells per mL of culture. Data were collected at 24-h point for 1 week, and viable cells were counted using trypan blue dye. **(B)** Cytotoxic evaluation of compounds in a dose-dependent manner was monitored by alamarBlue assay. **(C)** The effects of lead compounds on THb accumulation. The results are the average of three independent experiments and represented as mean ± SEM. * *p* < 0.05 was considered statistically significant when compared to untreated cultures.

The time-dependent cellular growth kinetic and cytotoxic effects of compounds **1** and **2** were determined by trypan blue and alamarBlue (AB) assay, respectively. The optimal active inducing doses of compound **1** (0.1 µM) and **2** (1 µM) showed no inhibitory effects on growth kinetics of cells, as compared to the untreated control. On the other hand, HU at 200 µM exhibited a sharp decrease in cell growth and proliferation with an increase in incubation time as shown in Figure 5A. The AB assay demonstrated dose-dependent anti-proliferative effects of lead compounds, expressed as a percent of cell viability of the untreated control. On one hand, Figure 5B indicates no inhibitory effect on cell viability of compound **1** at concentration <50 µM (IC_50_ = 5.431 µM) and for compound **2** at concentration <100 µM (IC_50_ = 12.39 µM). On the other hand, HU (200 µM) showed potent inhibition in cell proliferation at a concentration ≤ of 50 µM with IC_50_ = 72.46 µM. The prominent proliferation and non-toxic effects of compounds **1** and **2** provided proof of the safety and effectiveness of these compounds as potent erythroid differentiating agents at significantly lower doses with the least cytotoxic effects. For THb accumulation, treated K562 showed a significant amount of the total hemoglobin content (*p* < 0.05) when compared to untreated control cultures. Maximal THb production (23.22 ± 0.91 pg/cell) was observed with compound **1** administered for 5 days at 0.1 µM, while compound **2** at 1 µM exhibited higher contents of accumulated THb by 19.29 ± 0.10 pg/cell. These accumulated effects of compounds at optimal doses were even higher than the results obtained with 200 µM of HU (17.32 ± 1.18 pg/cell) as shown in Figure 5C.

### Fetal Hemoglobin Production Analysis

**FIGURE 6:**
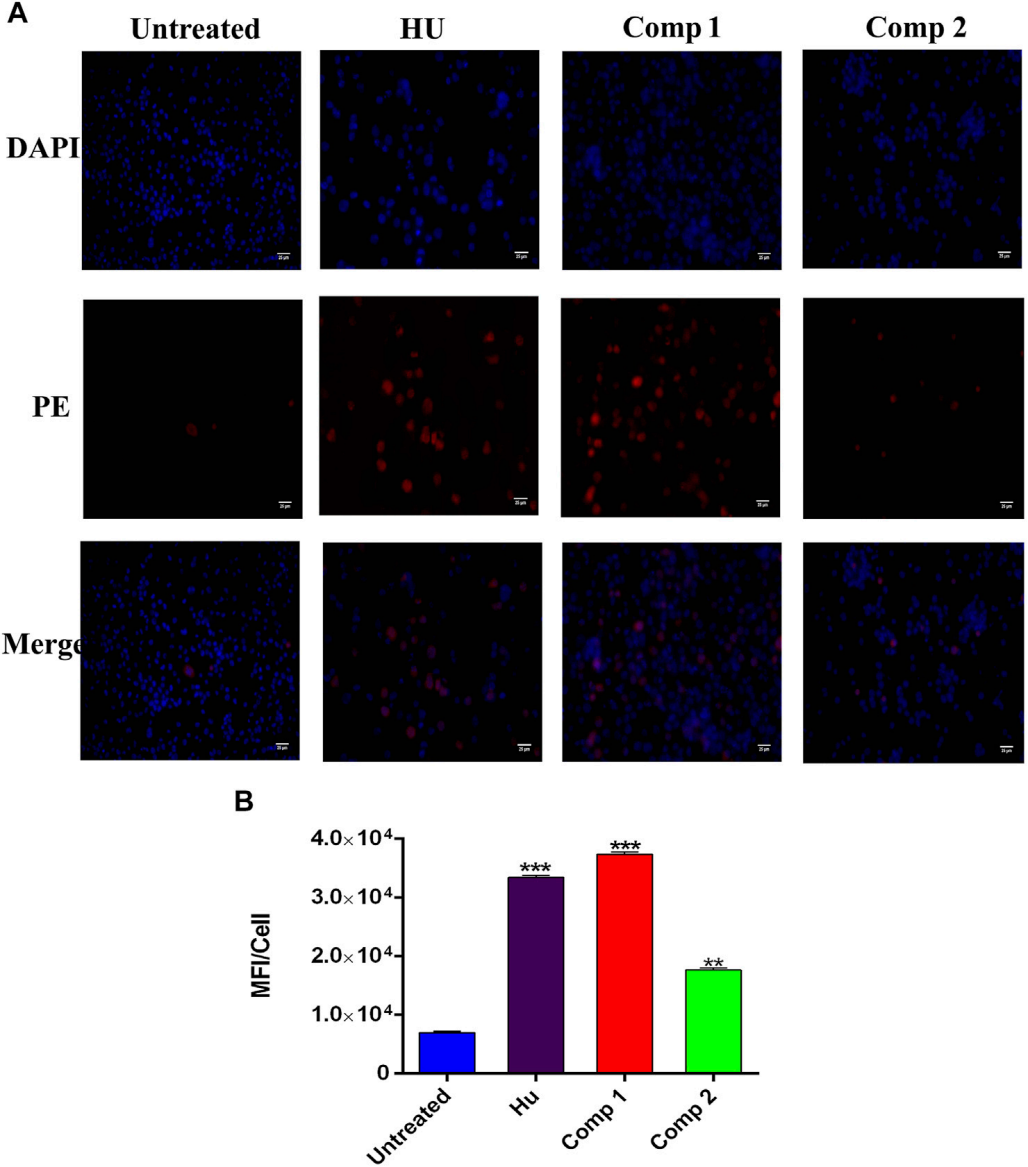
Immunofluorescence analysis K562 cells treated with compound **1** (0.1 µM), compound **2** (1 µM), and HU (200 µM) showed HbF expression in comparison to untreated cells. **(A)** The PE panel showed that treated compounds exhibited stronger fluorescent signals than the untreated culture. Cells were co-stained with DAPI for visualization and the pink color in the merge panel indicated the co-localization. (Scale bar = 25 µM). **(B)** Mean fluorescence intensity (MFI)/cells were calculated by ImageJ ver. 4.1.0. *p < 0.05 was considered statistically significant HbF expression in comparison to control.

The immunofluorescence (ICC) staining and flow cytometry analysis showed a marked-up rise in HbF levels induced by lead compounds, in comparison to HU and untreated cells. The ICC staining showed that compound **1** (0.1 µM) and compound **2** (1 µM) led to a great increase in HbF-positive cells stained with PE-conjugated anti-HbF antibodies when compared to the positive control HU (200 µM) as shown in Figure 6A. In control (untreated) cells, a low proportion of HbF-expressing cells was observed. To quantify ICC data, integrated densities per cell expressing HbF levels were calculated by using ImageJ software and are represented in Figure 6B.

**FIGURE 7:**
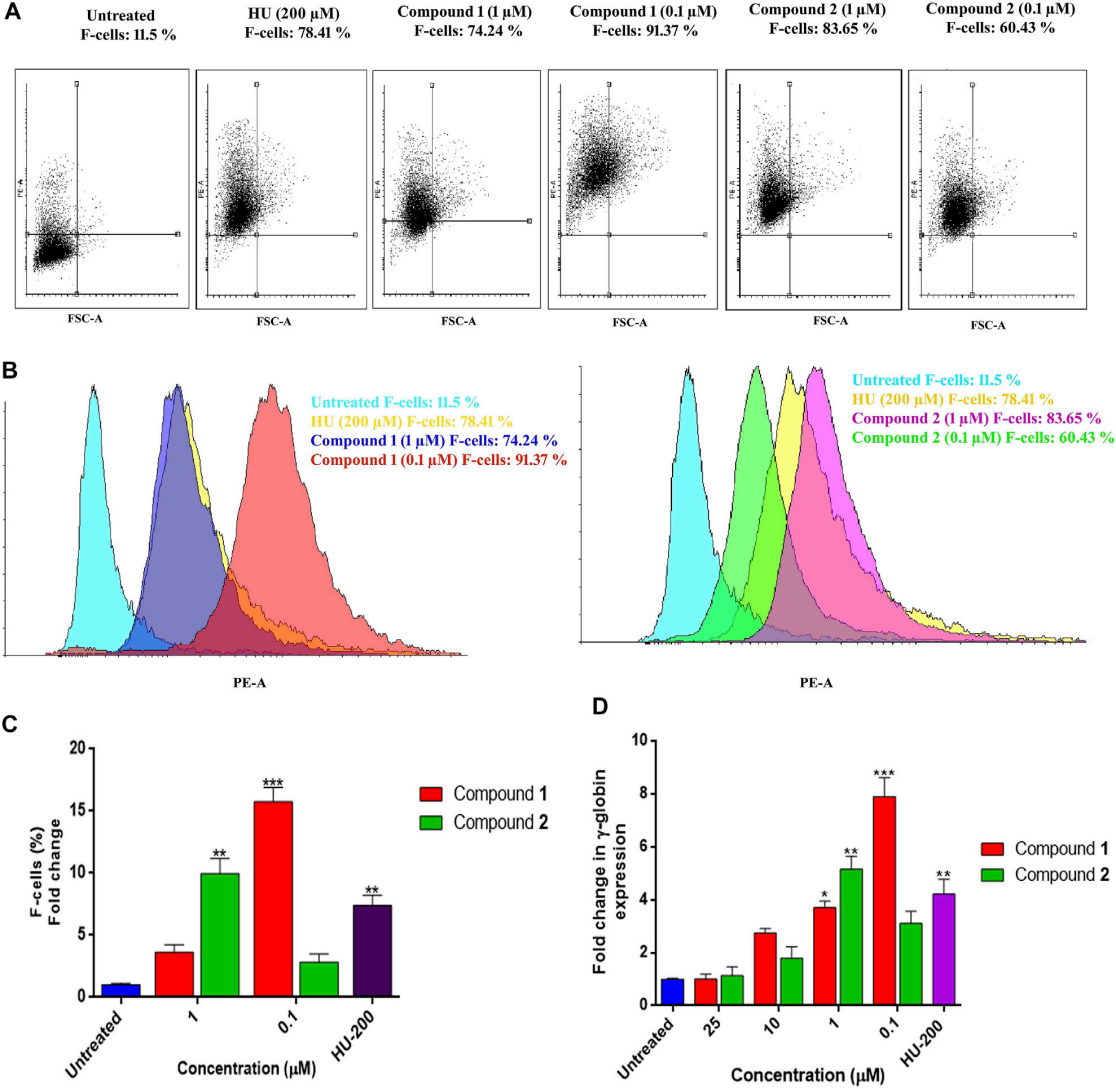
HbF expression analysis by flow cytometry and comparative fold change (qRT-PCR) analysis of γ-globin gene expression in K562 cells by lead compounds. **(A)** Dot plots depicting the HbF-positive cells stained with anti-HbF antibodies observed through the PE channel. **(B)** The histogram demonstrated the level of F-cells (HbF-containing cells) were upregulated by the treatment of lead compounds at different inducing concentrations. **(C)** The bar graph showed percentages of HbF-induced F cells in comparison to untreated and HU treated cultures. Data showed (mean ± SEM) are from three separate experiments. **(D)** The γ-globin expression level was normalized to the level of corresponding GAPDH. The significant differences (*p < 0.05) between compounds treated and the untreated control were analyzed by one-way ANOVA.

Similar results obtained with flow cytometry analysis are presented as dot plots in Figure 7A. K562 cells treated with inducing agents (**1)** and (**2**) at different concentrations showed concentration-dependent HbF expression of these compounds when compared to HU and the control. Figure 7B demonstrates compound **1** (0.1 µM) and compound **2** (1 µM) showed significant production of F-cells (HbF-positive cells) (91.37%) with 15.76 ± 1.14-fold increase (*p* < 0.001), 9.95 ± 1.23-fold change (*p* < 0.01), and 83.65% percent increase in F-cells when compared to non-treated cells, respectively. The individual effects of optimal inducing concentration of these compounds on K562 cells were even higher than the percentages of F-cells (78.41%) observed with HU treatment. The graphical representation of induced HbF-positive cells expressing increase in percentages and fold change of F-cells by lead compound treatment are presented in Figure 7C.

### Induction of γ-globin Gene Expression by Lead Compounds

The increased production of HbF-positive cells was found analogous to the results obtained with qRT-PCR analysis for γ-globin gene expression. An mRNA analysis on K562 cells for γ-globin gene expression treated with lead compounds in a concentration range of 0.1–25 µM for 5 days showed overexpression of the γ-globin gene. Compound **1** expressed an increase in HbF production at the gene level with a decrease in concentration as shown in Figure 7D. The fold change (1.06 ± 0.17) in the γ-globin gene with compound **1** at 0.1 µM was significantly increased to the level of 7.88 ± 0.72 –fold. Consequently, compound **2** also showed a dose-dependent increase in the production of the γ-globin gene. Compound **2** was able to significantly induce the γ-globin gene level with a fold change of 5.15 ± 0.78 at 1 µM.

### Computational Analysis Revealing Compound–Enzyme Interactions

**FIGURE 8:**
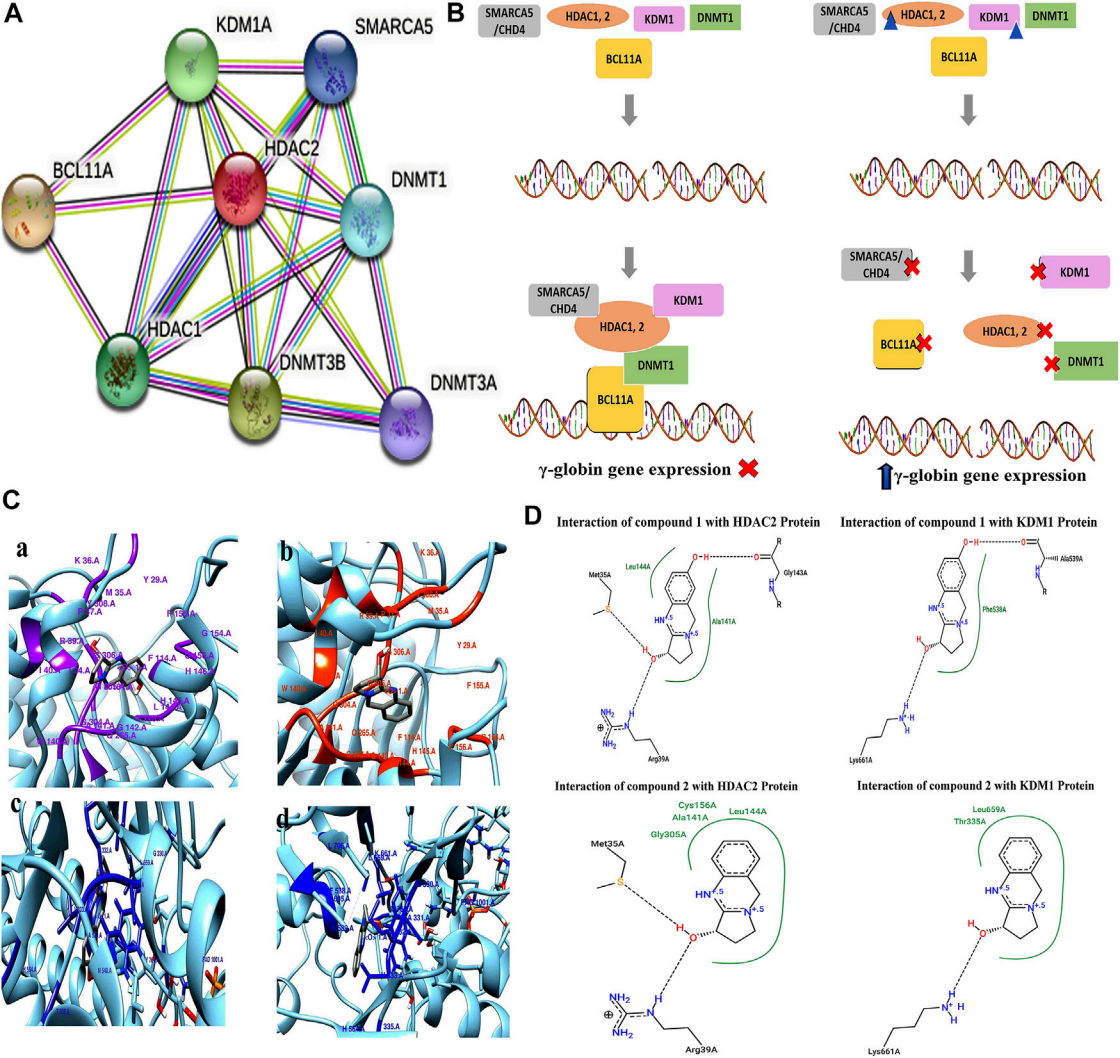
**(A)** STRING database analysis to generate PPIs of DNMT1, KDM1, HDAC1, HDAC2, and BCL11A proteins for γ-globin gene suppressor complex. **(B)** Proposed epigenetic mechanism indicating the inhibitory action of pure compounds (triangular shape) of *A. vasica* on HDAC2 and KDM1 proteins in the reactivation of the γ-globin gene. **(C)** 3D interaction generated through Chimera shows the interaction of compounds **1** and **2** with HDAC2 and KDM1 protein. **(D)** 2D schematic view generated through the PoseView tool for compound **1** showing interaction with HDAC2 protein through G143, L144, M35A, and R39A, while compound **2** depicts interaction with M35, R39, G305, N141, L144, and C156 of HDAC2. In KDM1 protein, compound **1** interact through L661, A539, and P538. Compound **2** shows interaction with L661, T335, and L659.

The protein–protein interaction (PPIs) network obtained from the STRING database recognized HDAC2 protein as a core protein as shown in Figure 8A. The corresponding interacting nodes of docked proteins were ranked according to the functioning score as presented in Supplementary Table S2. PPIs suggested that targeting all or one of these proteins could disrupt the whole complex of the γ-globin gene suppressor resulting in the γ-globin gene reactivation. The possible mode of action of these compounds that helps in the induction of the γ-globin gene is shown in Figure 8B.

The strong interaction of compounds **1** and **2** with KDM1 and HDAC2 proteins in terms of binding energies was found (Figures 8C,D). Among all proteins, compound **2** showed significant results against KDM1 and HDAC2 proteins with the binding affinity of ∼ −7.2 and ∼6.70 kcal/mol in comparison to hydroxyurea that resulted in −4.84 and −4.26 kcal/mol (Supplementary Table S3). The detailed 2D and 3D interactions of compounds with HDAC2 and KDM1 proteins are provided in the Supplementary Material.

## Discussion

β-thalassemia is caused by the inadequate production of adult hemoglobin (HbA, α2β2) due to reduced production of the β-globin chain of hemoglobin. Individuals before birth are usually asymptomatic because of the substantial level of fetal hemoglobin. The increased HbF production has proven to improve the severity and pathophysiology of the disease by compensating the globin chain’s imbalance caused by the absence of β-globin chains (Cui and Engel, 2017). To date, many HbF modulators have been identified, but their clinical effectiveness remains questionable (Fathallah et al., 2007; Ng and Ko, 2014). In this perspective, the identification of more pharmacological molecular entities that can induce HbF production to reduce the frequency of blood transfusion required for patients with β-thalassemia is still a necessity.

Considering the safety and efficacy of phytochemicals, many researchers have focused on bioactive phytochemicals with HbF-inducing potential. Chemical constituents of *A. vasica* have been known as herbal medicine and are pharmacologically important for the treatment of various ailments (Hossain and Hoq, 2016). However, no report has been found in the literature for HbF-inducing potential and activity-guided isolation of this multifaceted plant. Hence, the study was carried out on an *in vitro* model (human erythroleukemia K562 cells) following bioassay-guided isolation. K562 cells have been an extensively used model for the putative identification of new HbF inducers and to understand their kinetics and action mechanism involved in erythroid differentiation (Gambari, 2003).

In this study, preliminary screening of crude extracts of *A. vasica* has shown potent HbF-inducing ability in *in vitro* cultures as indicated by HbF-positive (green-colored) cells (Figure 1C) which was further validated at the molecular level in β-YAC transgenic mice. The results obtained from this *in vivo* study with IP administration of *A. vasica* for short and prolonged treatment in β-YAC transgenic mice indicated the pronounced HbF production and the upregulation of human γ-globin–encoding genes as compared to HU and control mice. Treatment with the aqueous extract of *A.vasica* at 100 mg/kg for four weeks induced γ-globin mRNA up to 5.59-fold above baseline in five different treated transgenic mice.

The *in vivo* safety of oral *A. vasica* was previously studied in Swiss albino mice; treatment with up to 1200 mg/kg of *A. vasica* given for 7 consecutive days for 4 weeks was found to be nontoxic and showed no adverse reaction on mice survival (Kumar et al., 2005). Similar observations were obtained from our sighting study to evaluate the LD50 of the aqueous extract of *A. vasica* using the OECD Test Guideline (TG) 425. Twenty mice (male and females) were randomly grouped into four groups (control and three test doses) of 5 mice each. The graded doses of the aqueous extract of *A. vasica* (1,000, 2,500, 5,000 mg/kg/bodyweight) and saline water (control) were orally administered to the mice. Cage-side sighting observations revealed the non-toxic nature of the plant extract that did not exhibit critical death effects to the mice and suggested the LD50 of the aqueous extract greater than 5,000 mg/kg.

This *in vivo* efficacy of *A. vasica* was supported by non-significant variations in hematological and biochemical indices at lower doses of *A. vasica*, thus signifying its safety in mice. The slight variations in WBC and platelet counts were observed at low doses of the plant that directed some mild effect on the hematological index. Moreover, significant variation in the total count, WBC, and platelet counts were also observed in a study by the administration of 200 and 500 mg/kg of *A. vasica* that might incline some serious effect on the hematological parameter.

Serum biochemical indices such as ALT, AST, AP, Bun, and creatinine levels are crucial indicators for hepatocellular and renal damages and their quantitative evaluation offers a degree of hepato-renal sensitiveness to a plant extract. The serum AP, AST, and ALT levels were found to be significantly altered by the intake of *A.vasica* (200 and 500 mg/kg), demonstrating impairment of the normal functioning of the liver. However, at 100 mg/kg of *A. vasica*, no major alterations were observed in biochemical indices indicating hepatoprotective effects and normal renal function of the plant at low doses. The outcomes of the study emphasized that the oral intake of up to 5,000 mg/kg of *A. vasica* in mice was safe for their survival. Furthermore, the hematological and biochemical indices obtained from the study have established the IP administration of 100 mg/kg of the extract of *A. vasica* was found to be safe and has no adverse effect on the functions of blood cells, the liver, and the kidneys in mice.

Based on the activity, four pyrroquinazoline alkaloids were isolated and characterized from a 30% methanol fraction of *A. vasica*. Two compounds (**1**) and (**2**) showed dose-dependent strong erythroid differentiation among the characterized compounds in the benzidine–Hb assay. Lead compounds elicit a comparable promising proliferating effect on cell viability and ample accumulation of THb contents than HU under the same culture conditions. The *in vitro* cultures treated with lead compounds (**1**) and (**2**) were able to significantly induce erythroid production expressing an increase in F-cells from 11% in untreated to 90% in *A. vasica*–treated cells. This increase in HbF production was consistent with an increase in γ-globin mRNA contents. Interestingly, pharmacokinetics and pharmacodynamics studies of *A. vasica* alkaloids have been well documented. These compounds have been known for their prolonged use without any serious health effects (Ahmad et al., 2009; Liu et al., 2013).

The protein–protein interaction and docking studies revealed that compounds have direct implications in the activation of γ-globin genes, which is supported by the high index of scoring among selected proteins and higher binding affinities of lead compounds with the DNA binding factor (BCL11A) and its recruited co-repressor complexes, which are involved in the silencing of HBG. The docking process was validated through a validation procedure that applies the known inhibitor of HDAC2 (LLX). Nonetheless, the AutoDock analysis for the predicted pathway needs further validation through MD Simulations, and confirmation through experimental setup is in consideration for the detailed mode of action of these compounds in HbF induction.

From these observations, we inferred the potential role of *A. vasica* and its major alkaloids in HbF-inducing ability, with normal physiological hematocrit counts and hemoglobin content *in vivo*, and hepato-renal protective effects of the extract explicating the non-toxic nature of *A. vasica*. However, in regards to HbF induction, further studies are still desirable for the exact mechanism involved in *A. vasica*-mediated γ-globin gene expression, differential globin genes expression profiling, promoter activity involved in individual globin gene switching and expression, key transcription factors involved in reactivation of HbF *in vivo*, and biochemical and hematological profiling with long-term administration (up to 6 months) in the *in vivo* mice model to fully anticipate the pharmacological HbF-inducing effects and safety of this plant in clinical utilization for hematological diseases.

## Conclusion

The study demonstrated the pivotal role of plant-based HbF inducers, based on the investigation of pharmacological agents that can stimulate HbF production. We present HbF-inducing activity-guided isolation of pyrroquinazoline alkaloids from *A. vasica* in a bioassay-guided manner. Compounds (**1**) and (**2**) showed significant reversal of the γ-globin silencing and encouraged the HbF production at lower doses without affecting cellular proliferation, supported by the evident increase in HbF production and γ-globin gene expression *in vitro*. The *in vivo* studies underline the therapeutic potential of *A. vasica* in fetal hemoglobin production at gene and protein levels with protective effects on blood cells and biochemical architectures, which supports the traditional use of *A. vasica* and would provide a great insight into the therapeutic use of *A. vasica* and pyrroquinazoline alkaloids in the treatment of hematological diseases.

## Data Availability

The raw data supporting the conclusions of this article will be made available by the authors, without undue reservation.
