# Supplementary material for: In Vitro and In Vivo Studies for the Investigation of γ-Globin Gene Induction by Adhatoda vasica: A Pre-Clinical Study of HbF Inducers for β-Thalassemia
Source: Front Pharmacol. 2022 Mar 29;13:797853. doi: 10.3389/fphar.2022.797853 (PMC9002120; doi:10.3389/fphar.2022.797853)
Supplement: Supplementary file 1 [file DataSheet1.docx]

**Supplementary data**

***In vitro* and *in vivo* studies for the investigation of γ-globin gene induction by *Adhatoda vasica*: A pre-clinical study of HbF inducers for β-thalassemia**

Fizza Iftikhar^a^, Saeedur Rahman^b^, Muhammad Behroz Naeem Khan^a^, Kanwal Khan^a^, Muhammad Noman Khan^b^, Reaz Uddin^a^ and Syed Ghulam Musharraf^a,b^ *

*^a^Dr.*Panjwani Center for Molecular*Medicine and Drug Research, International Center for Chemical and Biological Sciences, University of Karachi, Karachi-75270, Pakistan*

*^b^H.E.J. Research Institute of Chemistry, International Center for Chemical and Biological Sciences, University of Karachi, Karachi-75270, Pakistan.*

**Correspondence*: Prof. Dr. Syed Ghulam Musharraf

*Dr. Panjwani Center for Molecular Medicine and Drug Research, International Center for*

*Chemical and Biological Sciences, University of Karachi, Karachi-75270, Pakistan*

*E-mail address:*[musharraf1977@yahoo.com](mailto:musharraf1977@yahoo.com)

# 1. Spectroscopic Characterization of Compounds 1-4

**Compound 1:**  IR*V_max_* (KBr) cm^-1^: 3337 (O-H), 2925 (aliphatic C-H stretch), 3057 (aromatic C-H stretch) and 1689 (C=N stretch). EI-MS *m/z* (rel. abnd. %): 204 (85), 203 (100), 187 (20), 175 (30). LC-ESI-OTOF-MS/MS *m/z* (rel. abnd. %): (M+H) ^+^; 205.0986 (38), 187.0869 (100), 185.0716 (44), 169.0773 (21) and 159.0692 (12).

**Compound 2:** IR*V_max_* (KBr) cm^-1^: 3450 (O-H stretch), 2905 (aliphatic C-H stretch) 3099 (aromatic C-H stretch), 1687 (C=N stretch). EI-MS *m/z* (rel. abnd. %): 188 (100), 170 (12), 159 (22) and 116 (5). LC-ESI-QTOF-MS/MS *m/z* (rel. abnd. %): (M+H) ^+^; 189.1035 (39), 171.091 (100), 169.0772 (23), 154. 0772 (13), 143.092 (47) and 118.093 (37).

**Compound 3:** IR*V_max_* (KBr) cm^-1^: 3455 (O-H stretching), 3060 (Ar C-H stretching), 2930 (C-H stretching), 1690 (C=O stretching), 1630 (C=N stretching) and 1120 (C-O stretching). EI-MS *m/z* (rel. abnd. %): 218 (100), 201(19), 162 (60) and135 (30). LC-ESI-QTOF-MS/MS *m/z* (rel. abnd. %): (M+H) ^+^; 2019.1142 (50), 201.1036 (100), 186.0798 (22), 173.0519, 169.772 (39).

**Compound 4:** (IR*V_max_* (KBr) cm^-1^: 3320 (O-H stretch), 2923.5 (aliphatic C-H stretch), 3010 (aromatic CH stretch) and 1685 (C=O stretch). EI-MS *m/z* ((rel. abnd. %): 202 (70), 185 (25), 174 (15), 146 (100). LC-ESI-QTO-MS/MS *m/z* (rel. abnd. %): (M+H) ^+^; 203.0824 (20), 185.0715 (100), 183.0633 (15), 167.0609 (32) and 158. 0609 (10).

Table 1: ^1^H & ^13^C NMR chemical shifts values of compounds 1-4 in CD_3_OD.

| **Position** | **Compound 1**  *δ*_H_ (mult., *J* in Hz) *δ*_C13_ | | **Compound 2**  *δ*_H_ (mult., *J* in Hz) *δ*_C13_ | | **Compound 3**  *δ*_H_ (mult., *J* in Hz) *δ*_C13_ | | **Compound 4**  *δ*_H_ (mult., *J* in Hz) *δ*_C13_ | |
| --- | --- | --- | --- | --- | --- | --- | --- | --- |
| 1a | 3.74 (m, 1H) | 53.01 | 3.32 (m, 1H) | 47.51 | 4.25 (m, 1H) | 44.53 | 4.28 (m, 1H) | 44.59 |
| 1b | 3.60 (m, 1H) | --- | 3.44 (m, 1H) | --- | 4.00 (m, 1H) | --- | 4.00 (m, 1H) | --- |
| 2a | 2.12 (m, 1H) | 31.74 | 2.40 (m, 1H) | 30.77 | 2.63 (m, 1H) | 31.22 | 2.66 (m, 1H) | 31.09 |
| 2b | 2.06 (m, 1H) | --- | 2.19 (m, 1H) | --- | 2.16 (m, 1H) | --- | 2.18 (m, 1H) | --- |
| 3 | 5.10 (t, 1H, 8.0) | 73.47 | 4.66 (t, 1H, 6.4) | 72.53 | 5.08 (t, 1H, 8) | 73.04 | 5.12 (t, 1H, 7.8) | 73.26 |
| 3a | --- | 163.76 | --- | 164.75 | --- | 158.84 | --- | 161.84 |
| 4a | --- | 126.21 | --- | 132.42 | --- | 143.72 | --- | 150.47 |
| 5 | 6.99 (d, 1H, 8.8) | 121.39 | 7.18 (dd merged, 1H, 4.0) | 130.46 | 7.62 (d, 1H, 8.8) | 129.44 | 8.23 (dd, 1H, 8.2, 1.2) | 135.67 |
| 6 | 6.74 (dd, 8.8, 2.4) | 118.53 | 7.33 (dt, 8.0 and 4.0) | 128.34 | 7.31 (dd, 1H, 8.8, 2.8) | 110.05 | 7.83 (dt, 1H, 8.3, 1.4) | 128.00 |
| 7 | --- | 157.74 | 7.25 (dt, IH, 8.0 and 4.0) | 128.25 | --- | 158.06 | 7.54 (dt, 1H, 8.1, 1.2) | 127.16 |
| 8 | 6.60 (d, 1H, 2.8) | 116.22 | 7.13 (dd merged, 1H,) | 118.41 | 7.52 (d, 1H, 2.8) | 125.18 | 7.74 (dd merged 1H, 7.8) | 128.01 |
| 8a | --- | 121.19 | --- | 118.23 | --- | 122.91 | --- | 121.91 |
| 9 | 4.81-4.77 (dd each, 2H, 16.0) | 48.88 | 5.08 (t, 2H, 15) | 51.94 | --- | 162.36 | --- | 162.59 |

^1^H and ^13^C NMR Spectra of compound 1**
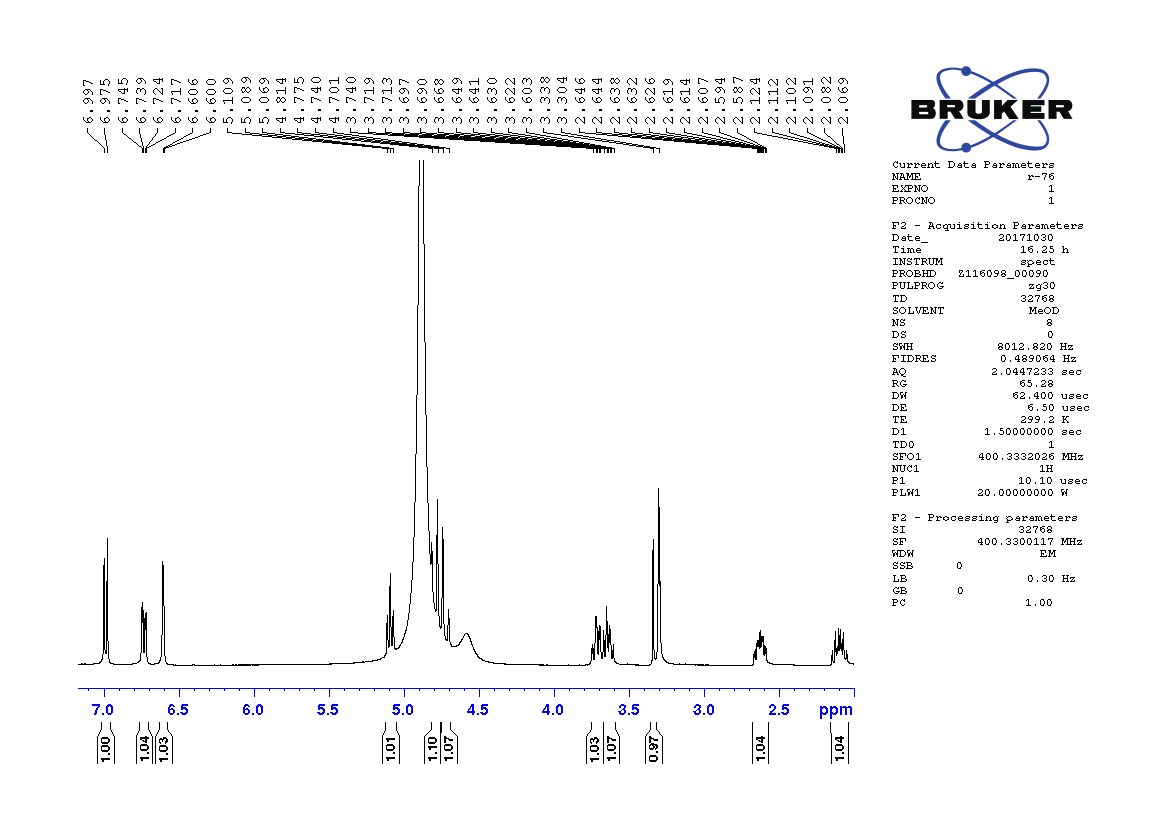
**


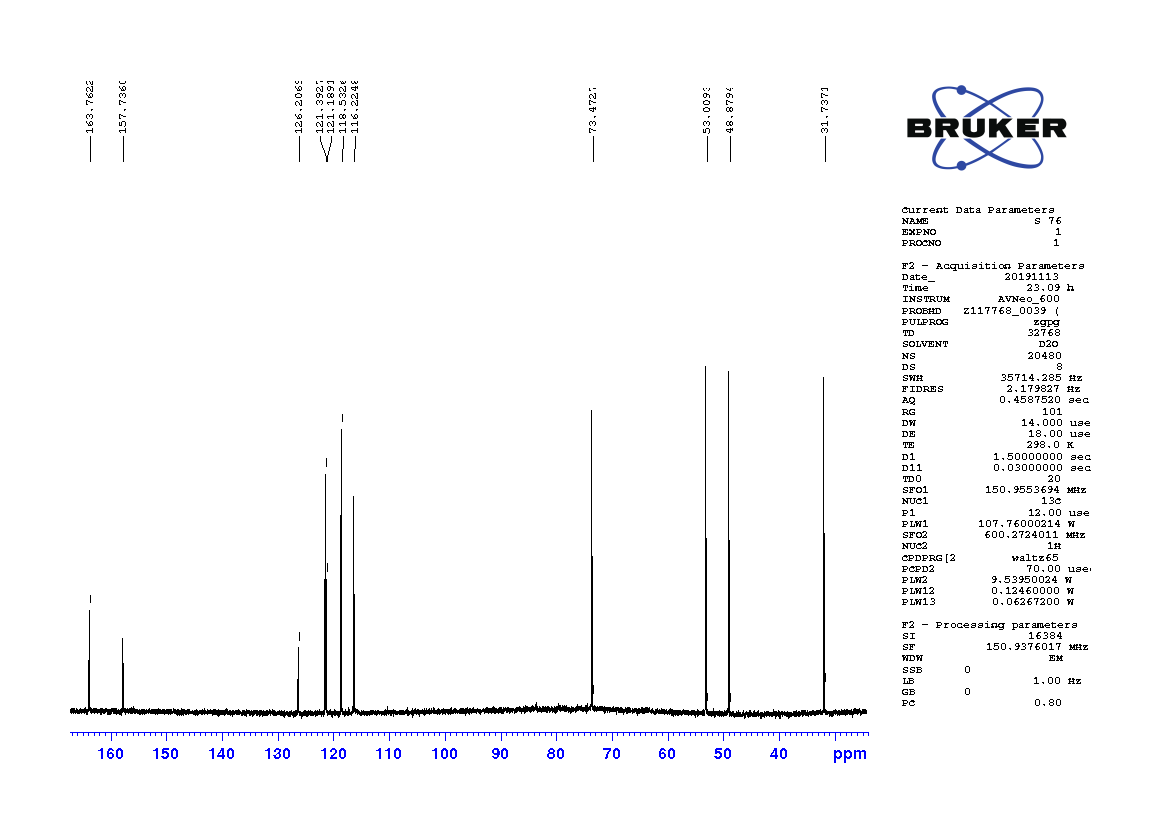


^1^H and ^13^C NMR Spectra of compound **2**
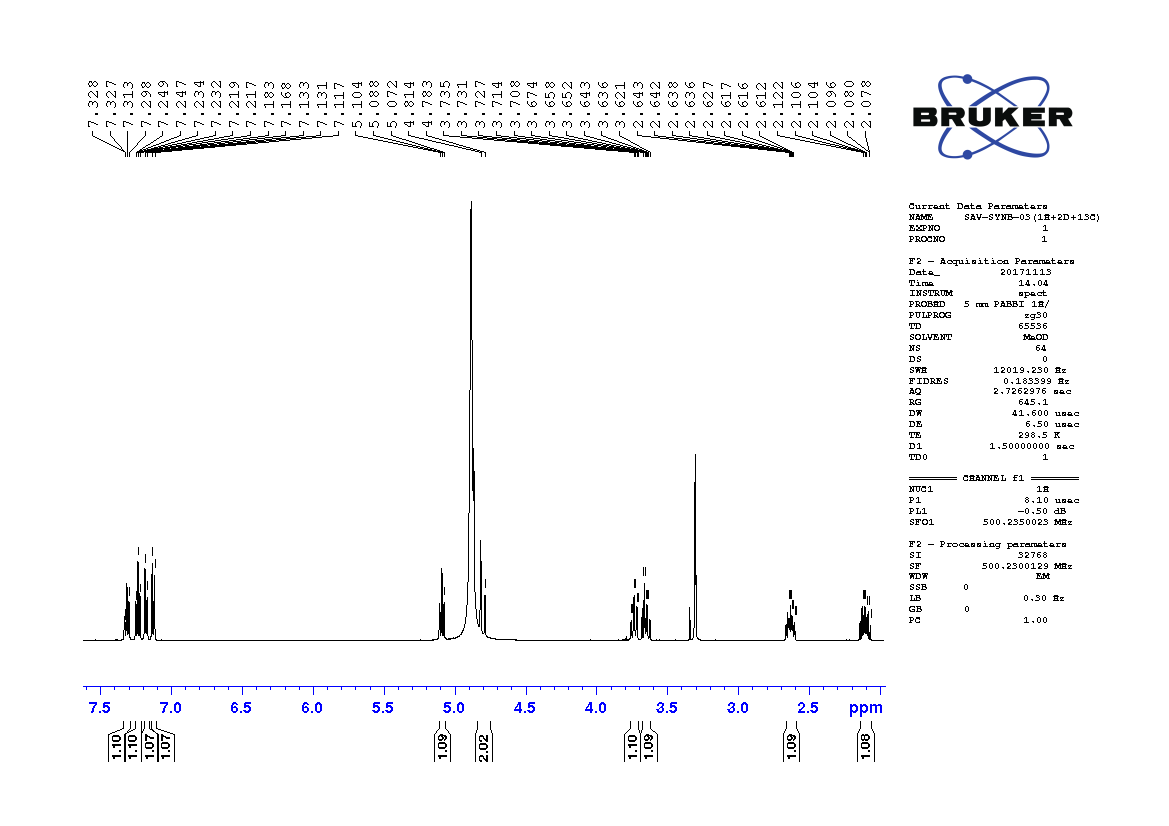


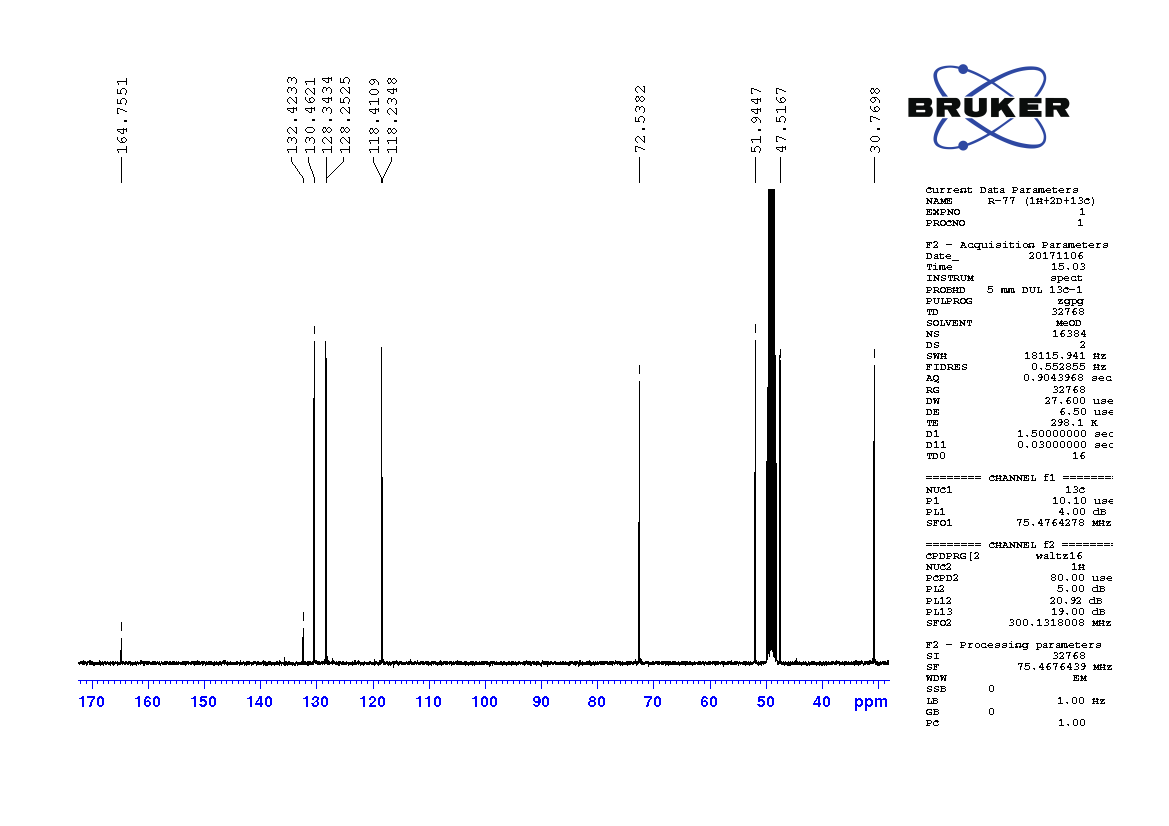


^1^H and ^13^C NMR Spectra of compound **3**


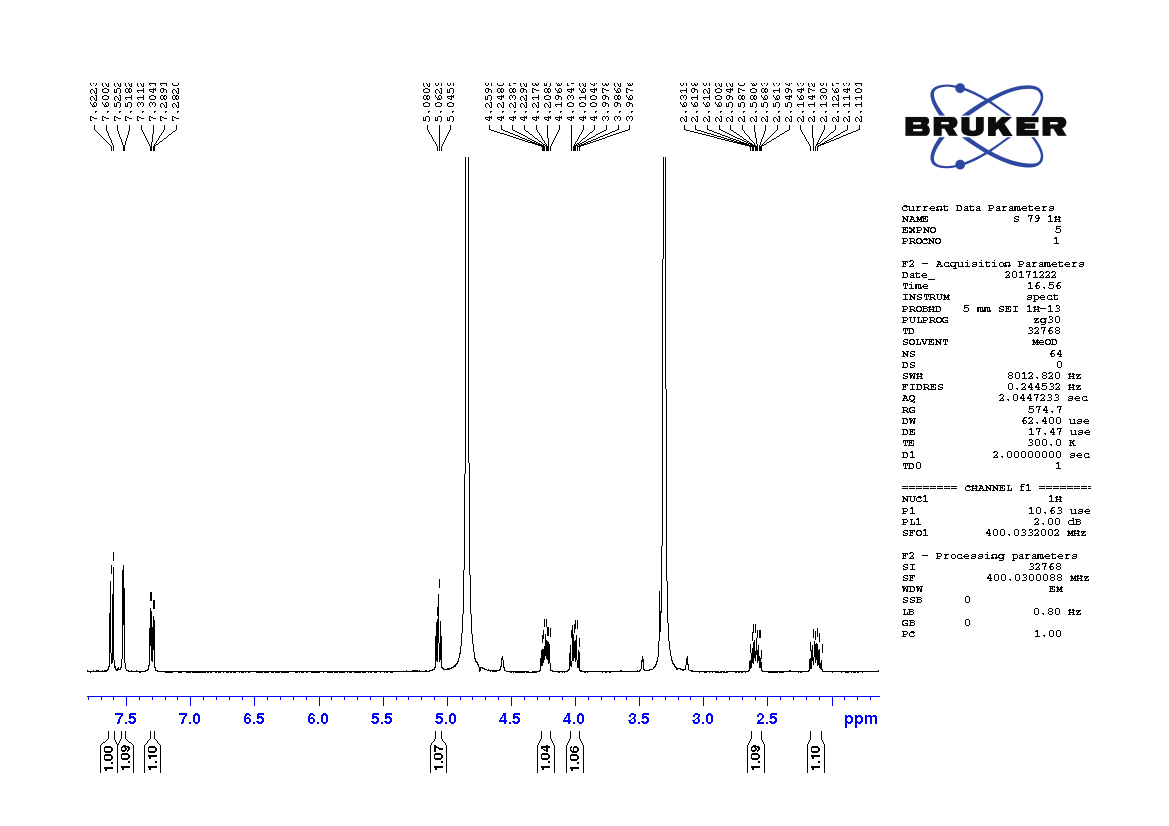


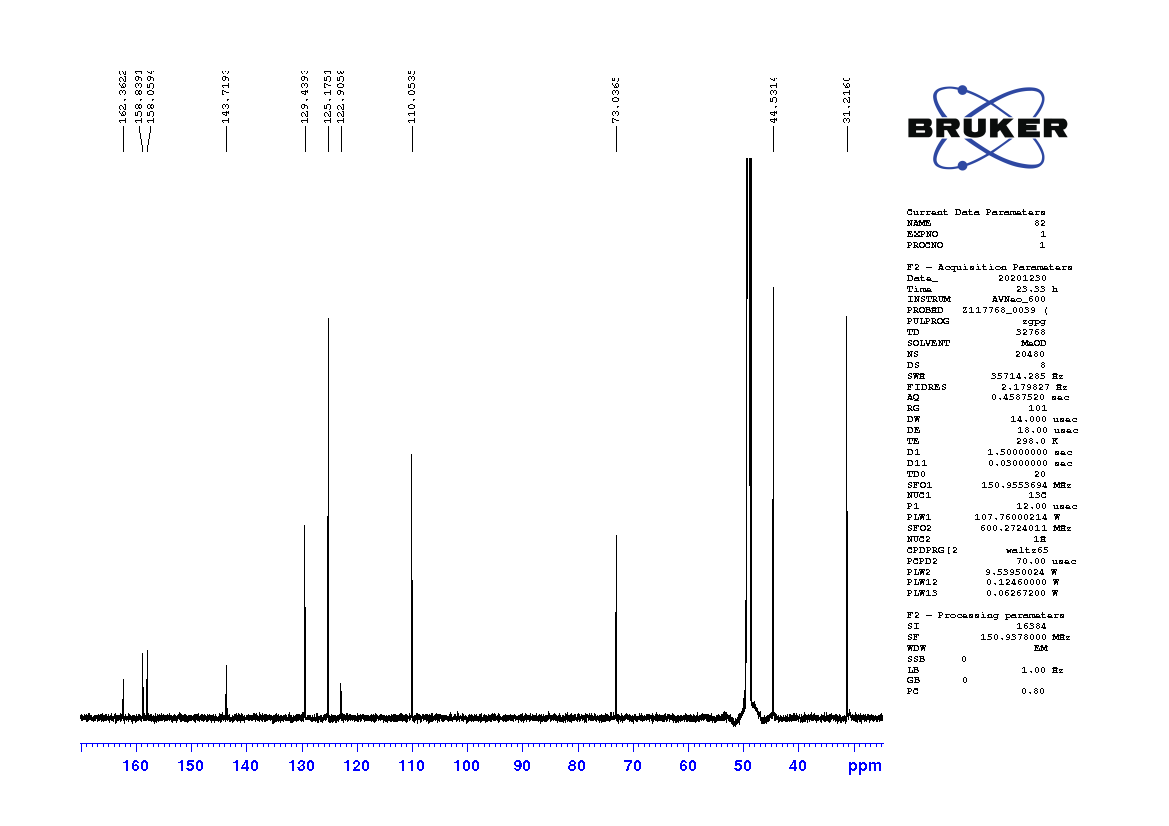


^1^H and ^13^C NMR Spectra of compound **4**


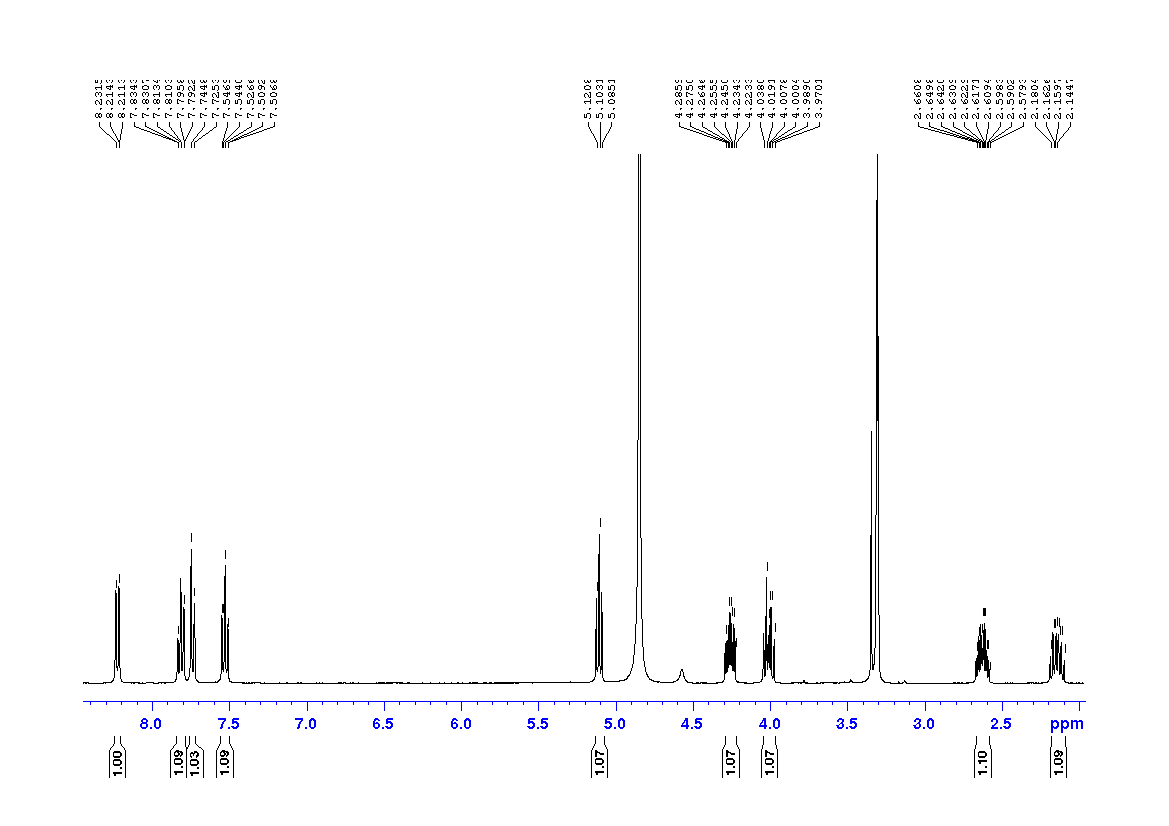


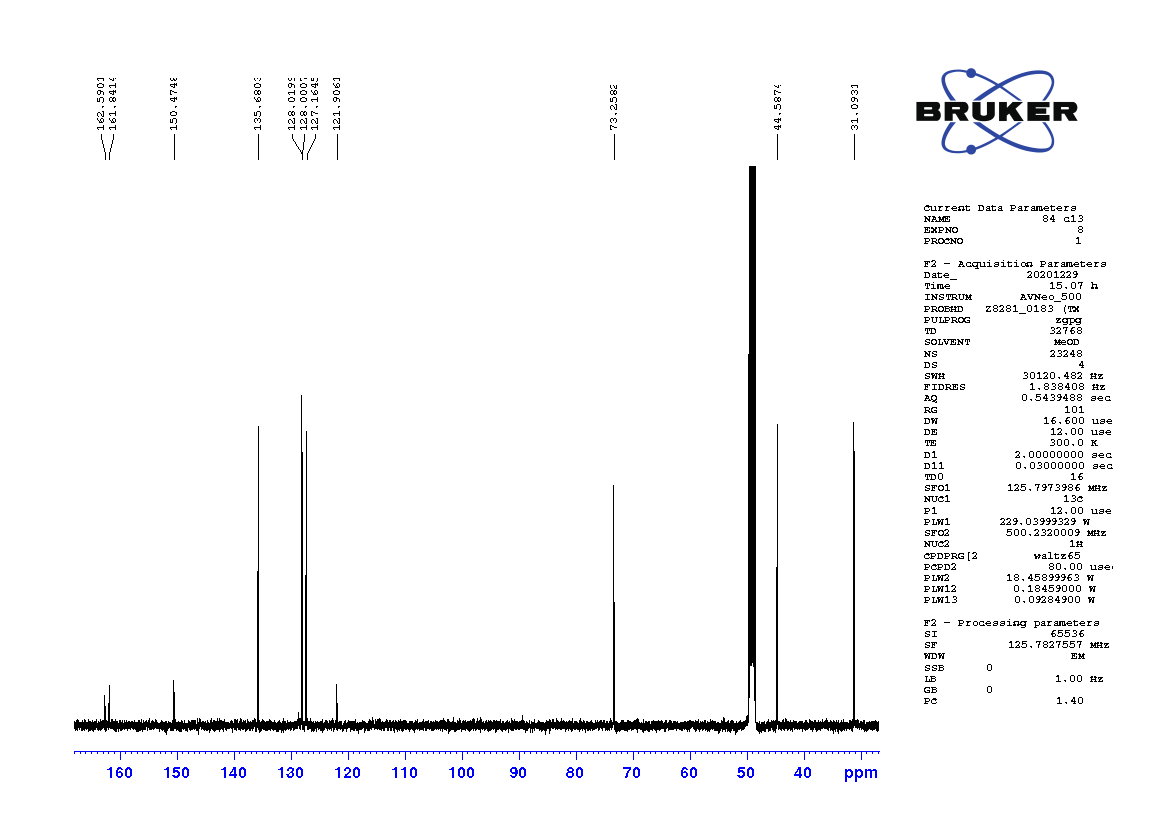


## 2. Molecular Docking Studies

## 2.1. Data Retrieval

The compounds used in the study were retrieved from PubChem. The 3D conformation was generated using a babel tool while energy minimization was performed through the FROG2 tool. Whereas, PDB database was used for the retrieval of Proteins i.e. DNA Methyltransferase (DNMT1) (PDB ID 4WXX), BCL11A (PDB ID 6KI6), HDAC2 (PDB ID 3MAX), HDAC1 (PDB ID 4BKX), and KDM1 (PDB ID 2DW4).

## 2.2. Data Retrieval for Validation Process

For the validation of docking and mode of action validation, two compounds were used i.e. hydroxyurea (known inducer of the gamma-globin gene with a different mode of action), LLX (N-(4-aminobiphenyl-3-yl) benzamide) (a known inhibitor of HDAC2), and FAD (coenzyme of KDM1). These compounds were retrieved from PDB with IDs of NHY, LLX, and FAD respectively.

## 2.3. Molecular Docking Studies

The standard docking procedure with parameters of 250 times Lamarckin GA setting resulting in 27,000 number of generation was used for the molecular docking using AutoDock 4.2 tool. The possible targeted interaction of DNMT1, KDM1, BCL11A, HDAC1 and HDAC2 (drug target) with respective ligands compounds **1** and **2** were studied through PoseView and the ezCADD tool.

## 2.4. Protein-Protein Interactions (PPIs) Studies

PPIs of these protein complexes were generated through STRING databases to highlight the interaction of these γ-globin gene suppressor proteins and to validate their function and interaction.

# 3. Active site Identification

The active site for targeted proteins was identified through a literature survey.

## 3.1. Active site Identification for BCL11A

BCL11A protein is a peptide, having an active site for DNA and Complex binding proteins, so the whole peptide was selected for docking studies.

## 3.2. Active site Identification for DNMT1

The active site having amino acids at position 1225(Cys), 1229(Ser), 1230(Gly), 1265(Glu), and 1311(Arg), was identified as an active site for DNMT1 against which ligands were docked.

## 3.3. Active site Identification for HDAC1

The residues in the active site pocket of HDAC1 comprise of Asp99, Asp176, Asp264, His 140, His141, His178, Phe150, Phe250, Lys271, and Tyr303.

## 3.4. Active site Identification for HDAC2

The amino acid at Arg39, His183, Gly305, Trp14, Gly142 sites were identified as active site residues against HDAC2 protein.

## 3.5. Active site Identification for KDM1

The active site having residues of Gly335, His812, Ala809, His364, and Thr810 was identified as an active site for KDM1.

# 4. Interaction of compounds with HDAC2 and KDM1

In HDAC2, compound **1** showed single hydrogen bond interaction with the hydroxyl group of Arg39 amino acid, while other hydrogen bonds were observed between the hydroxyl group of compound **1** and Gly143 amino acid. Thiol group was formed between Met35 and hydrogen of the hydroxyl group of compound **1**. Additionally, two hydrophobic interactions were found between Ala141 and Leu144 amino acids. Compound **2** also showed four hydrophobic interactions with Gly305, Al141, Cys156, and Leu144 through an aromatic ring, and one hydrogen bond was observed between Arg39 and (–OH) of compound **2**. While one thiol bond was also observed between (–OH) of compound **2** and sulfur of Met35. In KDM1, compound **1** showed one hydrogen bond interaction with Lys661, while the functional group (-OH) of compound **1** showed one hydrogen bond with Ala539. Also, one hydrophobic interaction was observed between Phe538 amino acid and compound **1** aromatic ring. Compound **2** also formed two hydrophobic interactions with Leu659 and Thr335 through an aromatic ring, while one hydrogen bond was observed between the Lys661 and the hydroxyl group of compound **2**.

Table 2: PPIs Interaction of transcriptional suppressor of the γ-globin gene.

|  | | | | | | |
| --- | --- | --- | --- | --- | --- | --- |
| **UniPort Protein ID** | **Gene Name** | **Average Node Degree** | **Clustering Coefficient** | **Interacting Partners** | **Annotation** | **Score** |
| **Q92769** | HDAC2 | 5.75 | 0.894 | BCL11A | B-cell lymphoma/leukemia 11A | 0.532 |
|  |  |  |  | DNMT1 | DNA (cytosine-5)-methyltransferase 1 | 0.986 |
|  |  |  |  | DNMT3A | DNA (cytosine-5)-methyltransferase 3A | 0.630 |
|  |  |  |  | DNMT3B | DNA (cytosine-5)-methyltransferase 3B | 0.977 |
|  |  |  |  | KDM1A | Lysine-specific histone demethylase | 0.985 |
|  |  |  |  | SMARCA5 | SWI/SNF-related matrix-associated actin dependent regulator of chromatin | 0.988 |
|  |  |  |  | HDAC1 | Histone deacetylase 1 | 0.955 |

Table 3: Binding free energy calculation results for DNMT1, KDM1, BCL11A, HDAC1, and HDAC2 bound with compounds 1 and 2 in comparison to HU*.*

| **Inhibitors** | **Binding Energy (Kcal/mol)** | | |
| --- | --- | --- | --- |
|  | Compound **1** | Compound **2** | HU |
| BCL11A | -4.75 | -4.68 | -4.16 |
| DNMT1 | -5.78 | -5.93 | -5.30 |
| HDAC1 | -5.57 | -5.27 | -4.77 |
| HDAC2 | -6.79 | -6.70 | -4.26 |
| KDM1 | -7.23 | -7.25 | -4.84 |
